# Observed crowding effects on *Mycobacterium tuberculosis* 2-*trans*-enoyl-ACP (CoA) reductase enzyme activity are not due to excluded volume only

**DOI:** 10.1038/s41598-017-07266-w

**Published:** 2017-07-28

**Authors:** Mariane Rotta, Luis F. S. M. Timmers, Carlos Sequeiros-Borja, Cristiano V. Bizarro, Osmar N. de Souza, Diogenes S. Santos, Luiz A. Basso

**Affiliations:** 1 Instituto Nacional de Ciência e Tecnologia em Tuberculose (INCT-TB), Centro de Pesquisas em Biologia Molecular e Funcional (CPBMF), Pontifícia Universidade Católica do Rio Grande do Sul (PUCRS), Porto Alegre, RS Brazil; 2 Programa de Pós-Graduação em Medicina e Ciências da Saúde, PUCRS, Porto Alegre, RS Brazil; 3 Laboratório de Bioinformática, Modelagem e Simulação de Biossistemas (LABIO), Faculdade de Informática, PUCRS, Porto Alegre, RS Brazil; 4 Laboratório de FarmInformática (FarmInf), Faculdade de Farmácia, PUCRS, Porto Alegre, RS Brazil

## Abstract

The cellular milieu is a complex and crowded aqueous solution. Macromolecular crowding effects are commonly studied *in vitro* using crowding agents. The aim of the present study was to evaluate the effects, if any, of macromolecular synthetic crowding agents on the apparent steady-state kinetic parameters (*K*_*m*_, *k*_*cat*_, and *k*_*cat*_ */K*_*m*_) of *Mycobacterium tuberculosis* 2-*trans*-enoyl-ACP (CoA) reductase (InhA). Negligible effects on InhA activity were observed for ficoll 70, ficoll 400 and dextran 70. A complex effect was observed for PEG 6000. Glucose and sucrose showed, respectively, no effect on InhA activity and decreased *k*_*cat*_ */K*_*m*_ for NADH and *k*_*cat*_ for 2-*trans*-dodecenoyl-CoA. Molecular dynamics results suggest that InhA adopts a more compact conformer in sucrose solution. The effects of the crowding agents on the energy (*E*_*a*_ and *E*_*η*_), enthalpy (*∆H* ^*#*^), entropy (*∆S* ^*#*^), and Gibbs free energy (*∆G* ^*#*^) of activation were determined. The *∆G* ^*#*^ values for all crowding agents were similar to buffer, suggesting that excluded volume effects did not facilitate stable activated *ES* ^*#*^ complex formation. Nonlinear Arrhenius plot for PEG 6000 suggests that “soft” interactions play a role in crowding effects. The results on InhA do not unequivocally meet the criteria for crowding effect due to exclude volume only.

## Introduction

The intracellular environment is a highly concentrated medium in which biological macromolecules, such as proteins, nucleic acids, ribononucleoproteins, and polysaccharides occupy a significant fraction (typically 20–30%) of the total volume^1, 2^. Since two molecules cannot occupy the same space in solution, the macromolecules will be mutually excluded from their neighbourhood, resulting in the excluded volume effects^3, 4^. In this crowded milieu, nonspecific interactions may result in changes in both thermodynamic parameters (e.g., decrease in entropy leading to increase in free energy of solute) and kinetics of enzyme-catalyzed chemical reactions^3, 4^. The increase in free energy resulting from volume exclusion can also be accounted for by an increase in the thermodynamic activity which, in turn, is due to a value larger than one for the activity coefficient^3^. Accordingly, there may be an increase in the rate of enzyme-catalyzed chemical reaction in the presence of crowding agents^3^. Molecular crowding can also affect chemical equilibrium, oligomerization, folding, protein-protein interactions, and conformational stability^3, 4^. The effects of crowding on biochemical reaction rates are complex and they are usually related to the nature of each reaction. In diffusion-limited reactions, crowding might reduce the overall rate by reducing diffusion^5^. If during catalysis there is an expansion of the transition state and its decay to product contributes to the rate-limiting step, molecular crowding may result in lower catalytic rates^3, 5^. Hence, efforts to evaluate the effects, if any, of excluded volume on enzyme-catalyzed chemical reactions should provide insights into attempts to extrapolate biochemical observations made *in vitro* to *in vivo*, which may inform efforts on screening of enzyme activity modulators.

The 2-*trans*-enoyl-ACP (CoA) reductase from *Mycobacterium tuberculosis* (InhA) is an enzyme that belongs to the Fatty Acid Synthase System II (FAS-II) that catalyzes the last elongation step in the mycolic acid biosynthesis^6, 7^. Mycolic acids are essential components of *M. tuberculosis* cell wall which have been associated with pathogen virulence^7^, the ability of mycobacteria to survive and replicate inside macrophages, and the inability of many antibacterial agents to cross the cell wall to gain access to the *M. tuberculosis* cytosol^6, 8^. It has been shown that InhA converts 2-*trans*-enoyl-ACP (or enoyl-CoA) substrates into acyl-ACP (or acyl-CoA) via a hydride transfer from the 4 *S* hydrogen of NADH (nicotinamide adenine dinucleotide, reduced form) co-substrate to the C3 position of the 2-*trans*-enoylthioesther substrate^9, 10^. This enzyme has been shown to be the primary target of isoniazid, a first-line anti-tuberculosis drug^11–13^, thereby validating InhA as a target for anti-tuberculosis drug discovery.

Owing to both the importance of this enzyme in *M. tuberculosis* physiology and as a “druggable” bona-fide target for the development of chemotherapeutic agents to treat tuberculosis (TB), evaluation of macromolecular effects on InhA mode of action was deemed worth pursuing. Since the excluded volume may affect the steady-state kinetic parameters of enzyme-catalyzed chemical reactions (*K*_*m*_, *k*_*cat*_ and *k*_*cat*_ */K*_*m*_), studies that mimic the intracellular crowded environment should provide relevant data for a better understanding of InhA mode of action *in vivo*. In this work, crowding agents with different sizes and chemical properties (ficoll 70 and 400, dextran 70, and PEG 6000) were employed in a concentration range of 25–200 mg mL^−1^ to evaluate the exclude volume effects on the apparent kinetic parameters of InhA. The activation energy (*E*_*a*_) for the InhA-catalyzed chemical reaction, the transition state enthalpy (*∆H* ^*#*^), entropy (*∆S* ^*#*^) and Gibbs free energy (*∆G* ^*#*^) were evaluated in the assay buffer (Pipes 100 mM pH 7.0). These parameters were compared with the experimental values obtained in the presence of 200 mg mL^−1^ of the crowding agents. Glucose (α-D-glucopyranose) was employed as negative control as it is as a component unit of sucrose, ficoll and dextran oligomers. Sucrose (α-D-glucopyranosyl-(1→2)-β-D-fructofuranose) was also used as it may be regarded as a component of ficoll oligomers. Molecular dynamics simulation was employed to try to explain the unexpected effects of sucrose on InhA-catalyzed chemical reaction. Moreover, as a nonlinear Arrhenius plot was observed for PEG 6000, “soft” interactions (attractive or repulsive) and, thereby, an enthalpic component, were invoked to explain these results.

## Experimental Section

### General

Crowding agents ficoll 70, ficoll 400, dextran 70, and polyethylene glycol (PEG) 6000 were purchased from Sigma. Other chemicals (including glucose and sucrose) and biochemicals were obtained from commercial sources without further purification. Stock solutions of the crowding agents were prepared in water. Pipes 100 mM (final concentration) pH 7.0 was used as the assay buffer and the addition of the crowding agent did not affect the pH of the solution. Control experiments were performed to rule out any non-catalyzed chemical reactions between the crowding agents and the substrates. For this purpose, the chemical assay (below) was carried out in the absence of InhA at saturating substrate concentrations. No linear decrease in absorbance was observed showing that any decrease in absorbance was due to InhA-catalyzed chemical reaction. To ensure that the crowding agents, or possible impurities they may contain, do not act as substrate of InhA, the enzymatic assay was performed in solutions containing the enzyme, crowding agents and saturating concentration of either NADH or 2-*trans*-dodecenoyl-CoA (DD-CoA). All crowders as well as negative control chemical compounds were tested at different concentrations (25, 50, 100, and 200 mg mL^−1^). No linear decrease in absorbance was observed showing that the crowding agents and negative controls used in this work do not act as substrates of InhA. Solutions were thoroughly mixed before data collection to rule out experimental artifacts.

### Expression and purification of 2-*trans*-enoyl-ACP (CoA) reductase

The expression and purification of the recombinant protein were performed as previously described^9, 14^. The DD-CoA substrate was synthesized and purified by reverse-phase HPLC using a 19 × 300 mm C_18_ µBondapak column (Waters Associates, Milford, MA) as previously described^10^. A value of 29,600 M^−1^ cm^−1^ was here determined for the InhA extinction coefficient at 282 nm following the protocol described elsewhere^15^. InhA concentration was thus determined spectrophotometrically at 282 nm by diluting 10 µL of purified enzyme to 500 µL of 100 mM Pipes (pH 7.0).

### Assay of enzyme activity

Recombinant enzyme activity was measured by a continuous spectrophotometric assay as previously described^14^, measuring the decrease in absorbance at 340 nm (ε = 6.22 × 10^3^ M^−1^cm^−1^) upon conversion of NADH and DD-CoA substrates into NAD^+^ and dodecanoyl-CoA products. The enzyme activity measurements were carried out in a UV-2550 UV/visible spectrophotometer (Shimadzu) equipped with a temperature-controlled cuvette holder, unless otherwise specified. The enzyme activity assays were performed at 25 °C in Pipes 100 mM pH 7.0 buffer. Apparent steady-state kinetic parameters were determined for both substrates from initial velocity measurements at varying concentrations of NADH (5–300 µM) and fixed-saturating DD-CoA concentration (105 µM), and at varying concentrations of DD-CoA (2.5–150 µM) and fixed-saturating NADH concentration (200 µM). All reactions were started with addition of 0.15 µM of recombinant InhA, and data collected at least in duplicates. Hyperbolic saturation curves of initial rate data were fitted to the Michaelis-Menten equation (Eq. 1), in which *v* is the initial velocity, *V* is the apparent maximum initial velocity, *A* is the varying substrate concentration and *K*_*m*_ represents the apparent Michaelis-Menten constant for the varying substrate^16^.

1 $$v=\frac{VA}{{K}_{m}+A}$$

The *k*_*cat*_ values were calculated from Eq. 2, in which *[E]*_*t*_ corresponds to the total concentration of enzyme subunits. Experimental data for apparent kinetic parameters for NADH are given in Table ST1.

2 $${k}_{cat}=\frac{V}{{[E]}_{t}}$$

The same experimental procedure was employed for initial rate measurements in the presence of different concentrations of the crowding agents and negative controls (25, 50, 100, and 200 mg mL^−1^). Initial velocity data as a function of increasing NADH concentrations were fitted to Eq. 1. Sigmoidal curves were obtained for the DD-CoA substrate and they were fitted to Eq. 3, in which *K*_*0.5*_ is a constant related to *K*_*m*_ that also contains terms related to the effect of substrate occupancy at one site on the substrate affinity of other sites, and *n* is the Hill coefficient (related to the index of cooperativity)^17^.

3 $$v=\frac{{V}_{\max }[S]}{{K}_{0.5}^{n}+[S]}$$

Owing to mixing limitations, the measurements for dextran 70 at 200 mg mL^−1^ were carried out using an Applied Photophysics SX.18MV-R stopped-flow spectrofluorimeter on absorbance mode. Experimental details for stopped-flow measurements are given in Supporting Information. The apparent kinetic parameters for DD-CoA are presented in Table ST1.

### Energy of Activation

The energy of activation (*E*_*a*_) of the InhA-catalyzed chemical reaction was determined in absence or in presence of either polymeric crowding agents or negative controls (200 mg mL^−1^) by measuring initial velocities in the presence of saturating concentrations of both NADH (200 µM) and DD-CoA (105 µM). The temperatures ranged from 15 to 35 °C (288.15–308.15 K) for the crowding agents and negative controls, except for PEG 6000 whose temperature interval was from 15 to 40 °C (288.15–313.15 K). InhA was incubated for several minutes at all temperatures tested and assayed under standard conditions to ascertain InhA enzyme stability.

The energy of activation (*E*_*a*_) was calculated from the slope (*E*_*a*_/*R*) of the Arrhenius plot fitting the data to Eq. 4, in which *R* is the gas constant (8.314 J mol^−1^ K^−1^) and *A* is the product of the collision frequency (*Z*) and a steric factor (*p*) based on the collision theory of enzyme kinetics^18^. Here, it is assumed a simplistic view to explain a complex phenomenon and that *A* is independent of temperature.

4 $$\mathrm{ln}\,{k}_{cat}=\,\mathrm{ln}\,A-(\frac{{E}_{a}}{R})\frac{1}{T}$$

The enthalpy (*∆H* ^*#*^), Gibbs free energy (*∆G* ^*#*^), and entropy (*∆S* ^*#*^) of activation were estimated using Eqs 5, 6 and 7, respectively, derived from the transition state theory of enzyme reactions^18^.

5 $${\rm{\Delta }}{H}^{\#}={E}_{a}-RT$$

6 $${\rm{\Delta }}{G}^{\#}=RT(\mathrm{ln}\,\frac{{k}_{B}}{h}+\,\mathrm{ln}\,T-\,\mathrm{ln}\,{k}_{cat})$$

7 $${\rm{\Delta }}{S}^{\#}=\frac{{\rm{\Delta }}{H}^{\#}-{\rm{\Delta }}{G}^{\#}}{T}$$

Energy values are in kJ mol^−1^, with *k*_*cat*_ in s^−1^, to conform to the units of the Boltzmann (*k*_*B*_: 1.3805 × 10^–23^ J K^−1^) and Planck (*h*: 6.6256 × 10^–32^ J s^−1^) constants, and *R* is as for Eq. 4. Errors on *∆G* ^*#*^ were calculated using Eq. 8^18^.

8 $${({\rm{\Delta }}G)}_{Err}=\frac{RT{({k}_{cat})}_{Err}}{{k}_{cat}}$$

To obtain an estimate of the activation energy for viscous flow of the liquid (*E*_*η*_), the viscosity of solutions containing 200 mg mL^−1^ of the crowding agents were determined at temperatures ranging from 15 to 40 °C (288.15 to 313.5 K) (Table ST2, Figure SF3). All measurements were performed in duplicates. The relationship between viscosity and temperature is also described by the Arrhenius plot, and data were thus fitted to Eq. 4, in which *k*_cat_ is replaced with *η*, the solution viscosity, and *E*_a_ is replaced with *E*_*η*_, which may be regarded as the activation energy to viscous flow of the liquid (an energy barrier that must be surmounted in order for a molecule to “squeeze” by its neighbours if it is to undergo transport in the bulk medium). It should be pointed out that the *E*_a_ in Eq. 4 and Eq. 5 represents the energy of activation of the chemical reaction catalyzed by InhA including the energy for viscous flow (*E*_*η*_).

### Density and viscosity measurements

The determination of density and viscosity were performed using a Viscometer SVM 3000 (Anton Paar) at 25 °C. All measurements were carried out in duplicates (Table ST1). To calculate the volume fraction of crowding agents in aqueous solution, the partial specific volumes were estimated as previously described (Table ST1 and Figure SF1)^19, 20^. In short, values of the density of the solution containing the crowding agent (ρ in g cm^−3^) were plotted as a function of increasing solute concentration (c in g cm^−3^ or g mL^−1^), yielding a straight line (Figure SF1) from which the partial specific volume (υ in cm^3^ g^−1^ or mL g^−1^) could be determined (Eq. 9). ρ_0_ represents the density of the solvent in the absence of crowding agent.

9 $$\rho ={\rho }_{0}+(1-\nu {\rho }_{0})c$$

### Fluorescence spectroscopy

The binding, if any, of sucrose to free InhA enzyme was evaluated by fluorescence spectroscopy^14^. Protein fluorescence measurements were carried out at 25 °C by making microliter additions of sucrose (5–103.5 mg mL^−1^) to 2 mL of 2 μM InhA in Pipes 100 mM pH 7.0 keeping the dilution to a maximum of 1.1%. The sucrose stock solution concentration was 3 M. Larger sucrose concentration values could not tested due to solubility limitations of the stock solution. Excitation wavelength was 297 nm and the emission wavelength range was 310 ≤ λ ≤ 400 nm. The excitation and emission slits were 1.5 and 5 nm, respectively. Fluorescence determinations were performed using a RF-5301PC Spectrophotometer (Shimadzu). InhA was incubated for several minutes before the assay to avoid temperature effects on fluorescence measurements. Two control experiments were carried out: 1) microliter additions of sucrose (5.14–94 mg mL^−1^) to 2 mL of Pipes 100 pH 7.0, and 2) microliter additions of water (10–200 μL) to 2 mL of 2 µM InhA in Pipes 100 mM pH 7.0. The protein fluorescence data were fitted to Eq. 10, in which *F*_*o*_ is the observed fluorescence signal intensity of sucrose titration into a solution containing InhA (2 μM) in Pipes 100 mM pH 7.0, and *F* is the protein fluorescence signal of addition of water to a solution containing InhA (2 μM) in Pipes 100 mM pH 7.0 buffer. The ratio [EL]/[[E]_T_ represents the fraction of enzyme-ligand binary complex in comparison to total enzyme subunit (binding sites) concentration. As the maximum fluorescence intensity values in the emission spectra were at 338 nm, this wavelength was chosen to record the fluorescence intensity to measure, if any, binary complex formation.

10 $$\frac{[EL]}{{[E]}_{T}}100=\frac{{F}_{0}-F}{{F}_{0}}100$$

### Molecular Dynamics simulations of InhA in sucrose and glucose solutions

To evaluate the crowding effect of sucrose on the tetrameric biological unit of InhA, three different macromolecular systems were built: (1) InhA in water, (2) InhA in a water solution containing 25 mg mL^−1^ of sucrose, and (3) InhA in a water solution containing 200 mg mL^−1^ of sucrose. As starting conformation, the crystal structure of InhA (PDB ID: 1P44 chain A)^21^ was employed after removing the ligands NADH and inhibitor. All systems were enclosed in an orthorhombic box extended 10.0 Å from the protein surfaces. Molecular dynamics (MD) simulations were performed using ff99SB^22^ and GLYCAM-06EP^23^ force fields implemented in Amber14^24^. All MD simulations were computed in a NPT ensemble at 298.16 K with the Berendsen temperature coupling^25^ and constant pressure of 1 atm, with isotropic molecule-based scaling. The SHAKE algorithm^26^ was applied with a tolerance of 10^−5^ Ǻ to fix all bonds that contained a hydrogen atom, allowing the use of a 2.0 fs time step in the integration of the equations of motion. Periodic boundary conditions were applied, with electrostatic interactions between non-bonded atoms evaluated with the CUDA version of the particle-mesh Ewald (PME) method^27^. The Lennard-Jones interactions were evaluated using a 9.0 Ǻ atom-based cutoff^28^. To better accommodate the solvent molecules around the protein solute, all systems were submitted to a relaxation phase in which the protein structure was gradually released from a restriction potential of 20 kcal mol^−1^ to free motion in 400 ps. After heating and equilibration, the systems proceeded in the production phase for another 10.0 ns with no reassignment of velocities. Snapshots were collected at every 5.0 ps for analysis. Images were prepared with QtiPlot^29^ and PyMOL^30^. Similar MD simulation protocols were employed for glucose solutions. The hydrodynamic radius of tetrameric InhA (28.8 Å) in the absence of crowding agents was calculated using AMBER package^24^.

## Results

### Crowding effects on apparent steady-state kinetics parameters for NADH substrate

**Figure 1:**
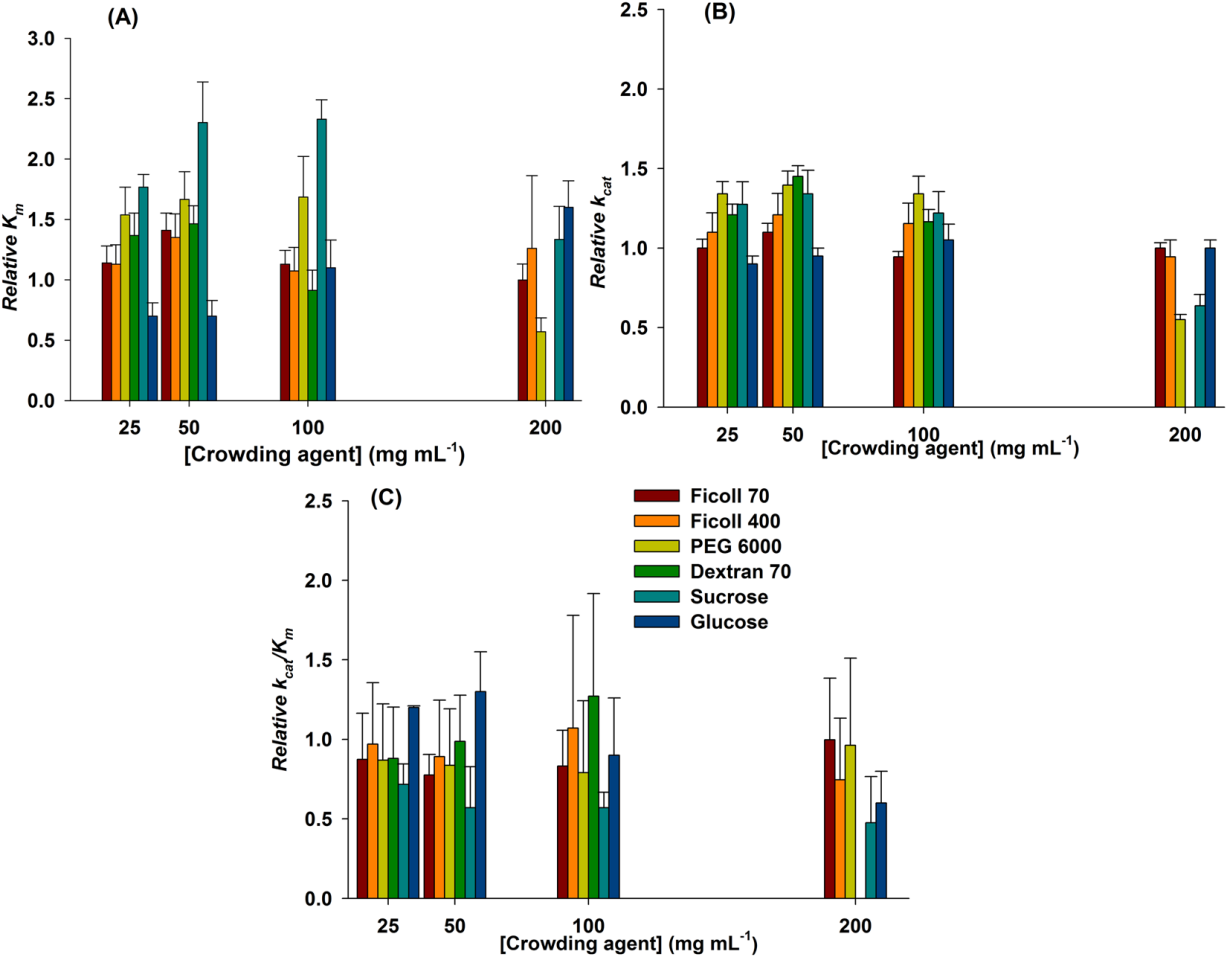
Relative apparent kinetic parameters determined for NADH substrate. (**A**) Relative apparent *K*_*m*_ values obtained for NADH substrate; (**B**) Relative apparent *k*_*cat*_ values obtained for NADH substrate; (**C**) Relative apparent *k*_*cat*_ */K*_*m*_ values obtained for NADH substrate. Error values are also reported in Supporting Information, Table ST1.

The apparent steady-state kinetics parameters for InhA were first determined in the absence of crowding agents. The values obtained for *K*_*m*_, *k*_*cat*_, and *k*_*cat*_ */K*_*m*_ in Pipes 100 mM were similar to those previously reported^9, 10, 14^. No changes in the apparent kinetic parameters for NADH were observed in the presence of increasing concentrations of ficoll 70 and ficoll 400 (Fig. 1, Tables ST1 and ST2).

Although a non-linear dependence of *K*_m_ and *k*_*cat*_ values was observed for increasing concentrations of dextran 70 (Fig. 1), the apparent second-order rate constant (*k*_*cat*_/*K*_m_) values appeared not to have been affected (Tables ST1 and ST2). Despite the fact that dextran 70 has been used at higher concentrations (>100 mg mL^−1^) in previous work^31^, in our hands, no reliable data could be obtained at 200 mg mL^−1^ of this background agent even using a stopped-flow apparatus for rapid mixing and measurement.

Non-linear effects were also observed for PEG 6000 as crowding agent when compared with diluted solution of Pipes 100 mM. The *K*_*m*_ values (Fig. 1, Table ST1) at 25, 50, and 100 mg mL^−1^ were, respectively, 1.5 (173 ± 26 μM), 1.6 (187 ± 26 μM) and 1.7 (190 ± 38 μM) times larger than Pipes 100 mM (113 ± 39 μM). There was an increase of approximately 1.3 times for *k*_*cat*_ (∼12 s^−1^) at these PEG 6000 concentrations as a value of 9 s^−1^ was obtained for a solution not containing this crowding agent (Fig. 1, Table ST1). Although lower *K*_m_ and *k*_*cat*_ values were obtained at 200 mg mL^−1^ (1.5-fold decrease), the *k*_*cat*_/*K*_m_ values were fairly similar for all concentrations of PEG 6000 (Fig. 1, Table ST1). The *K*_*m*_ for NADH increased 1.8 (199 ± 12 μM), 2.3 (259 ± 38 μM) and 2.3 (263 ± 18 μM) fold for sucrose solutions at, respectively, 25, 50 and 100 mg mL^−1^ (Fig. 1, Table ST1). No effects were observed for *k*_*cat*_ values at 25, 50 and 100 mg mL^−1^ of sucrose. A 1.5-fold decrease in *k*_*cat*_ value (5.8 ± 0.5 s^−1^) was observed at sucrose concentration of 200 mg mL^−1^. The *k*_*cat*_/*K*_m_ values decreased as a function of increasing sucrose concentration (Fig. 1, Tables ST1). Glucose has no effect on *k*_*cat*_ values for NADH (Fig. 1, Table ST1). Pearson correlation analysis was carried out to detect statistically significant linear correlations between the apparent steady-state kinetic parameters of InhA and increasing concentrations of crowding agents (Table ST2). The decrease in *k*_*cat*_/*K*_m_ value at 200 mg mL^−1^ glucose concentration (Fig. 1, Table ST1) showed no significant statistical correlation (Table ST2). Statistical analysis of data showed that no significant and tight correlation could be found for K_m_, *k*_*cat*_ or *k*_*cat*_/K_m_ values for NADH in sucrose nor for any kinetic parameter for both substrates in glucose (Table ST2).

### Crowding effects on apparent steady-state kinetic parameters for DD-CoA substrate

**Figure 2:**
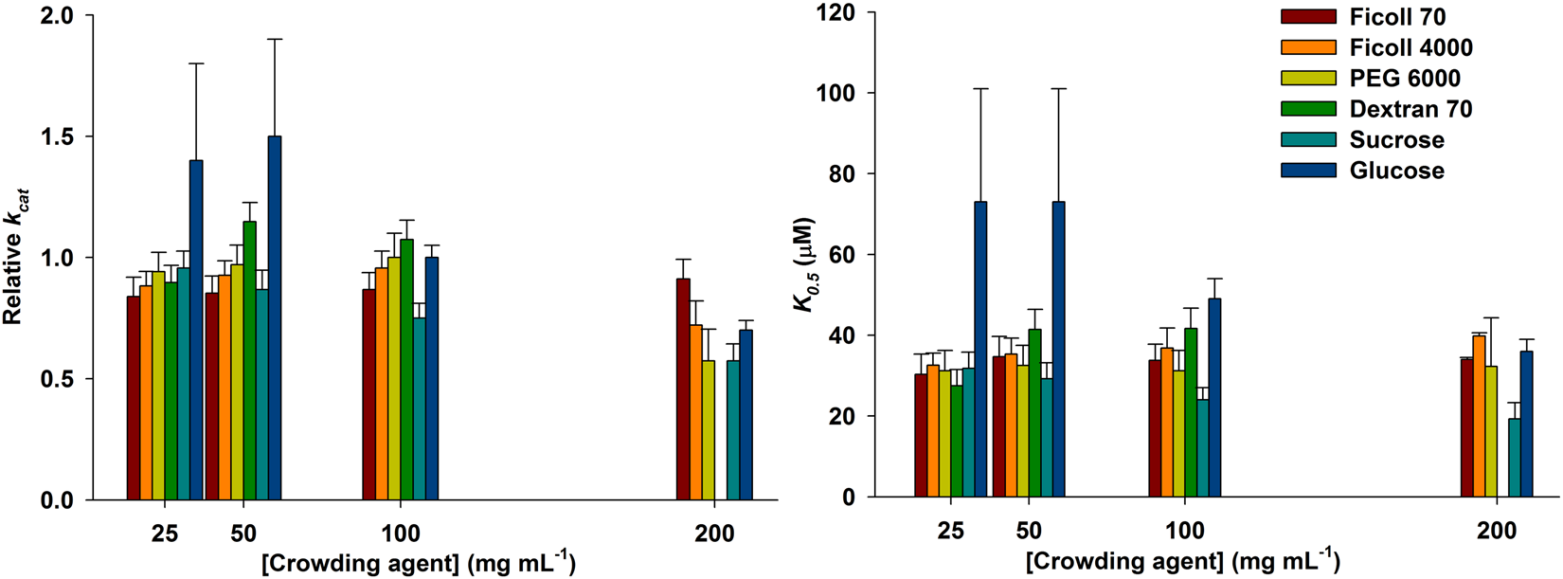
Relative apparent kinetic parameters determined for DD-CoA substrate. (**A**) *K*_*0.5*_ values obtained for DD-CoA substrate in the presence of crowding agents; (**B**) relative *k*_*cat*_ values obtained for DD-CoA substrate in the presence of crowding agents. Error values are also given in Table ST1.

In the absence of crowding agents, the initial velocity measurements for DD-CoA substrate were hyperbolic indicating Michaelis-Menten kinetics. However, the addition of background particles resulted in a sigmoidal profile for DD-CoA (Figure SF4). As the *K*_*0.5*_ constant does not equate to *K*_m_, a straightforward comparison is not warranted. All values for *K*_*0.5*_, *n*, and *k*_*cat*_ are given in Table ST1. Within experimental error, the *K*_*0.5*_ values observed were fairly similar for all crowding agents tested, except for sucrose that showed a decrease in *K*_*0.5*_ values as the concentrations of this crowding agent increased (Fig. 2A). Fluorescence spectroscopy measurements suggested no intermolecular interaction between sucrose (up to 103.5 mg mL^−1^) and free InhA (2 μM) (Figure SF5). Accordingly, the sigmoidal profile for sucrose solutions appears not to be due to sucrose interaction with NADH and DD-CoA binding sites of InhA. The decrease in *K*_*0.5*_ values for glucose solutions were both not systematic and fairly within experimental errors. A highly significant (P = 0.0006) and tight correlation (r = −0.9936) between increasing concentrations of sucrose and decreasing *k*_*cat*_ values for DD-CoA was observed (Table ST2). A similar trend was also found in *K*_0.5_ values for DD-CoA in sucrose (r = −0.9797; P = 0.0203), but not for glucose (Table ST2).

Comparing to Pipes 100 mM, no effects on *k*_*cat*_ for DD-CoA substrate were observed for ficoll 70 (r = −0.1247; P = 0.8417) and dextran 70 (r = 0.5110; P = 0.4890) (Fig. 2B, Tables ST1 and ST2). Again, in our hands, no reliable data could be obtained for dextran 70 at 200 mg mL^−1^. Interestingly, at the largest concentrations (200 mg mL^−1^) of ficoll 400, PEG 6000 and sucrose lower *k*_*cat*_ values for DD-CoA were observed (Fig. 2B, Table ST1). A pattern of decreasing *k*_cat_ values as a function of increasing sucrose concentrations was observed (Fig. 2B). Although glucose appears to lower *k*_*cat*_ values for DD-CoA, no clear trend could be observed for this effect (Fig. 2B, Table ST1). As mentioned above, Pearson correlation analysis shows that highly significant (P = 0.0006) and tight correlation (r = −0.9936) was observed between increasing concentrations of sucrose and decreasing *k*_*cat*_ values for DD-CoA (Table ST2), but not for glucose.

### Crowding effects on thermodynamic activation parameters for InhA

The energy (*E*_*a*_), enthalpy (*∆H* ^*#*^), entropy (*∆S* ^*#*^), and Gibbs free energy (*∆G* ^*#*^) of activation for InhA-catalyzed chemical reaction were estimated in diluted buffer (Table 1). The energy of activation to viscous flow of the liquid (*E*_*η*_), the energy barrier that molecules have to overcome to move in the bulk medium, was also determined in diluted buffer (Table ST2 and Figure SF3). The values obtained were compared to the results in the presence of background agents and are described in Table 1. As the cytosol of many types of cells has been reported with macromolecular occupation typically between 20–30% of the total volume^1^, the concentration of 200 mg mL^−1^ was selected for this experiment. As mentioned above, dextran 70 at 200 mg mL^−1^ concentration yielded no reliable data and was thus not evaluated.

**Table 1:** Thermodynamic activation parameters for InhA.

Parameter	Crowding agents^a^						
	Pipes 100 mM	Ficoll 70	Ficoll 400	PEG 6000		Sucrose	Glucose
*E*_*a*_ (kcal mol^−1^)^b^	9.9 ± 0.21	10.4 ± 0.2	10.12 ± 0.8	21.66 ± 0.4	7.64 ± 1.75	14.1 ± 0.4	12 ± 1
*E*_*η*_ (kcal mol^−1^)^b^	−4.0 ± 0.1	−5.09 ± 0.08	−5.22 ± 0.05	−5.46 ± 0.06		−4.7 ± 0.1	−4.9 ± 0.2
*∆H* ^*#*^ (kcal mol^−1^)^b^	5.4 ± 0.1	4.8 ± 0.2	4.3 ± 0.3	15.6 ± 0.3	1.5 ± 0.3	8.8 ± 0.2	6.7 ± 0.6
*∆S* ^*#*^ (cal mol^−1^ K^−1^)^b^	−36.6 ± 0.9	−38 ± 2	−40 ± 3	−3.0 ± 0.1	−50 ± 11	−26.3 ± 0.6	−32 ± 3
*∆G* ^*#*^ (kcal mol^−1^)^b^	16.2 ± 0.1	16.1 ± 0.1	16 ± 1	16.5 ± 0.1	16.5 ± 0.04	16.4 ± 0.05	16.2 ± 0.3

^a^Crowding agents at 200 mg mL^−1^. ^b^Values determined at 25 °C (298.15 K).

**Figure 3:**
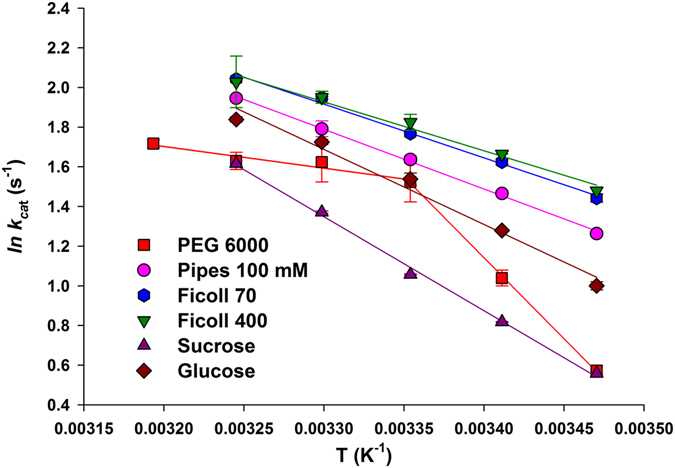
Arrhenius plot for different crowding agents (temperature dependence of ln*k*_cat_). Saturating NADH (200 µM) and DD-CoA (105 µM) concentrations and 200 mg mL^−1^ of crowding agents were used to determine the maximum velocity as a function of temperature ranging from 15 to 35 °C for crowding agents, except for PEG 6000 (15 to 40 °C).

The Arrhenius plot for Pipes 100 mM, ficoll 70, ficoll 400, sucrose and glucose showed a linear relationship between *ln(k*_*cat*_ *) versus* T^−1^ values (Fig. 3). On the other hand, a more complex profile was observed for PEG 6000, making the data analysis nontrivial (Fig. 3). All measurements showed a *∆G* ^*#*^ value of approximately 16 kcal mol^−1^. Lower *∆H* ^*#*^ values were observed for ficoll 70 and 400 in comparison to Pipes 100 mM, whereas the *E*_*a*_ and *∆S* ^*#*^ values were within experimental error (Table 1). Sucrose and glucose solutions showed larger *E*_*a*_ and *∆H* ^*#*^, and *∆S* ^*#*^ values. Finally, owing to the nonlinear Arrhenius plot for PEG 6000 (Fig. 3), two set of values for *E*_*a*_, *∆H* ^*#*^, *∆S* ^*#*^ and *∆G* ^*#*^ were calculated (Table 1)^16, 18^. Interestingly, lower temperatures showed larger *E*_*a*_, *∆H* ^*#*^ and *∆S* ^*#*^ values, while the opposite holds for increasing temperatures. At any rate, there is enthalpy-entropy compensation as the *∆G* ^*#*^ values were all similar.

To try to evaluate whether or not there is any nonlinear function between viscosity and temperature, the activation energy for viscous flow of the liquid (*E*_*η*_) was determined measuring the viscosity of solutions containing 200 mg mL^−1^ of the crowding agents at various temperatures (Table ST2, Figure SF3). All crowding agents displayed a linear pattern (Figure SF3), with negative *E*_*η*_ values (Table 1). These data are consistent with a lowering of the energy barrier that must be surmounted in order for a molecule to “squeeze” by its neighbours if it is to undergo transport in the bulk medium at increasing temperatures. The linear Arrhenius plot suggests that there are no gross changes in physical chemical properties of PEG 6000 solutions in the temperature range here described. For instance, aggregation of PEG 6000 polymeric chains at a particular temperature could result in change in viscosity that could be detected by a nonlinear Arrhenius plot. The nonlinear pattern observed for PEG 6000 is thus a property of the enzyme-catalyzed chemical reaction in this crowded solution and not a nonlinear property of viscosity of this crowding agent.

### Molecular Dynamics (MD) of InhA in sucrose and glucose solutions

**Figure 4:**
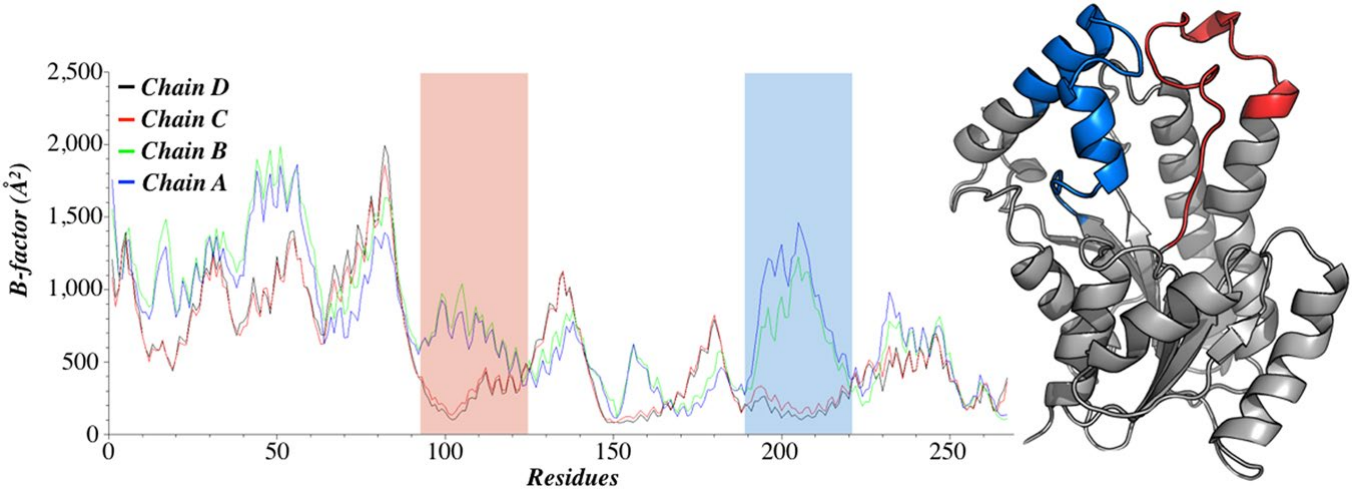
B-factor for each of the subunits of tetrameric InhA during the simulation at sucrose concentration of 25 mg mL^−1^. The two regions showing the largest variations among the subunits A/B and C/D were highlighted. These same regions are colored in the three dimensional structure on the right side. Image prepared with QtiPlot^29^ and PyMOL^30^.

MD simulations show both differences in the flexibility of InhA and variation in the volume of the active site. These alterations could be related to the concentrations of sucrose (25 mg mL^−1^ and 200 mg mL^−1^) employed to try to mimic the crowding effect on InhA enzyme activity. MD results show that InhA assumes a more rigid form at larger concentrations of sucrose (200 mg mL^−1^) when compared to the simulations with 25 mg mL^−1^ and without the crowding agent, which is borne out by the B-factor variance for each subunit in all simulations (Figure SF6). In addition, when the protein surroundings is not fully saturated with the crowding agent (25 mg mL^−1^), the subunits show variations in their flexibilities. Figure 4 shows the B-factor for each of the four subunits of InhA in the simulation with 25 mg mL^−1^ sucrose. The B-factor describes the displacement of the atomic positions from their average, which is indicative of the local dynamics within a protein. Therefore, low B-factors values indicate rigid parts, whereas large values indicate flexible regions. In short, B-factor values typically reflect protein motions - the more mobile a part of the protein, the higher the B-factor values. Interestingly, the most affected region is the loop motif defined by helices α6 and α7, which defines the substrate-binding site in subunits C and D (Fig. 4).

**Figure 5:**
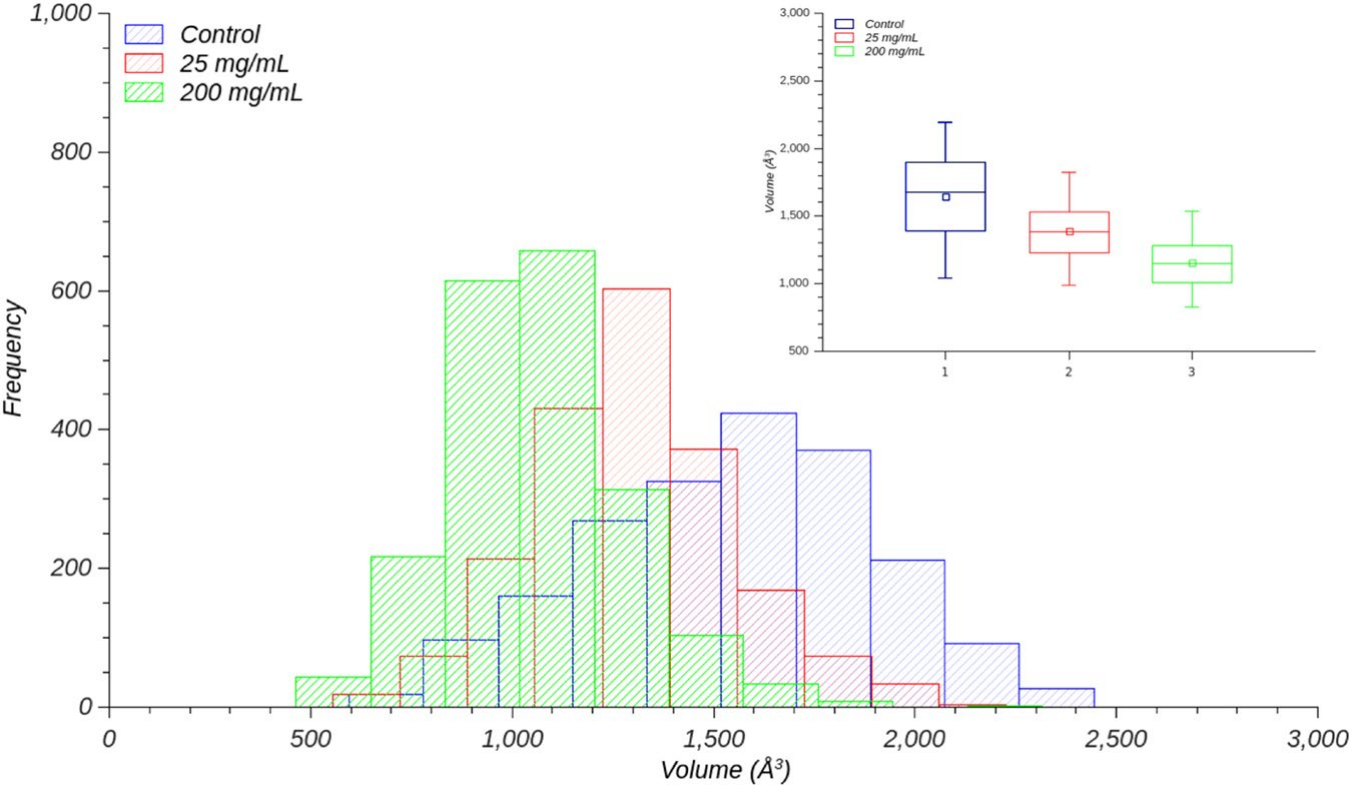
Distribution of the volume of InhA active site cavity for subunit A. The bars for the simulation at sucrose concentration of 200 mg mL^−1^ are colored in green, in red is given the simulation for sucrose at 25 mg mL^−1^, and the control is shown in blue. Top right inset presents a box plot with the mean and variance of the values to all systems. Image prepared with QtiPlot^29^.

The MD simulations were also carried out at 200 mg mL^−1^ sucrose concentration. The results show lower B-factor values and variations in the volume of InhA active site cavity (Figure SF6, panel C; Figure SF7). The MD results suggest that subunit A adopts a more compact form at 200 mg mL^−1^ concentration of sucrose as compared to both lower concentrations of this crowding agent or in its absence (Fig. 5). A fairly similar pattern can be observed for subunits B and D as compared to subunit A, except subunit C that appears not to be influenced by sucrose (Figure SF7). Notwithstanding, when all subunits are analyzed, it can be observed that most of the values assessed by subunit C are in the same interval described for the others subunits (Figure SF8). Moreover, there appears to be a unimodal density function with a lowering of the volume of active site cavity of InhA in solutions containing large sucrose concentration (Figure SF9). Interestingly, the control simulation, without sucrose, has a normal distribution having structures in closed and open conformations (Figure SF9). As InhA has been shown to be a homotetramer in solution^32^, the more compact structure of subunit A (Fig. 5) and the similar pattern observed for subunits B and D suggest that the overall protein structure is more compact even if subunit C has not undergone the same structural rearrangement (Figure SF7, panel C). It was deemed appropriate to show the distribution of volumes of subunit A as it gives a clear description of the overall results. Accordingly, it is tempting to suggest that InhA active site packing could interfere with processes such as substrate entrance and/or product release.

MD simulations were also carried out in glucose solution at 200 mg mL^−1^ concentration. The volume of the InhA substrate-binding cavity (InhASBC) in the solution containing glucose (200 mg mL^−1^) was compared with InhASBCs for sucrose at 200 mg mL^−1^ and PIPES 100 mM. One-way analysis of variance (ANOVA) with RStudio was employed to assess whether there are any statistically significant differences between their means by the Games-Howell (GH) test at 95% confidence level (Table ST4). The GH test was preferred because the Levene’s statistics revealed that the variance of our distributions are unequal (*p* < 2.2 × 10^–16^). The null hypothesis (*H*_*0*_) was: control group has the same average volume as the treatment groups. The alternative hypothesis (*H*_*A*_) was: control group has a different average volume from the treatment groups. In addition, given the independence of the dynamic behaviour of each subunit, comparisons of all subunits in PIPES 100 mM (control), sucrose and glucose solutions were performed (Table ST4). Comparisons between control (PIPES 100 mM) and the sucrose group at 200 mg mL^−1^ showed *p-values* < 0.01 for subunits A, B, and D, indicating that there is a statistically significant difference between the means of these distributions. For subunit C, the *p-value* = 0.14 denotes no significant difference between the means. Comparisons between control (PIPES 100 mM) and the glucose group at 200 mg mL^−1^ yielded *p-values = *1.00 for subunits A and C, indicating no significant differences between their means. For subunits B and D, with *p-values* < 0.01, the differences between the means are statistically significant. These results suggest that the volume of InhASBC can distinguish the different experimental systems, in which the control group (PIPES 100 mM) is more similar to glucose at 200 mg mL^−1^ than to sucrose solution at the same concentration. Overall, the results from MD simulations corroborate the hypothesis that the InhA enzyme exhibits a more compact form in sucrose- than in glucose-containing solutions.

## Discussion

As the excluded volume effect should be a result of non-specific interactions between the test protein and the background particles, it has been proposed that an experimental approach using different sizes, shapes, and numbers of crowding agents should be pursued^31^. The following criteria have been proposed to be useful for discriminating between crowding effects and specific interactions between the test protein and background particles^33^: (*i*) observe similar effects with different crowding agents (to demonstrate the lack of significant interaction between protein and crowder); (*ii*) there should be a disproportionate increase in the phenomenon being measured with increasing background agent concentration; (*iii*) large crowding agents should cause greater effects than small particles when compared at the same number density; (*iv*) small crowding agents should cause greater effects than large particles when compared at the same weight concentration. Accordingly, neutral hydrophilic polysaccharides ficoll 70 and 400 were used to evaluate whether there is any effect of size of the crowding agent on the apparent steady-state kinetic constants of InhA. The effects of chemically different crowding agents on the kinetic constants of InhA were evaluated employing another neutral polysaccharide, dextran 70, and the neutral polymer PEG 6000. All these crowding agents have compact and largely spherical shape, which results in a small surface to volume ratio^4^. Moreover, their neutral and relatively hydrophilic nature should minimize specific interactions with proteins^4^. PEG, ficoll, and dextran are thus believed to act primarily via excluded volume effect by decreasing the effective volume available and, thus, increasing the effective protein concentration^4^. It has been reported that amongst effective conditions for studying macromolecular crowding effects, the test molecule and crowder should have similar sizes^4^. Therefore, the knowledge of the hydrodynamic radii of the crowding agent and the test molecule is important for selection of suitable experimental conditions. Accordingly, ficoll 70 and PEG 6000 were employed as their hydrodynamic radii are somewhat similar to tetrameric InhA. Sucrose was employed because both it is a cosolute having similar polarity to a solution of either ficoll 70 or dextran 70 at the same concentration and it is the monomeric unity of ficoll 70^34^. Glucose was employed as negative control as it is as a component unit of sucrose and, therefore, of ficoll and dextran oligomers. All these agents were evaluated at 25–200 mg mL^−1^ concentration range because the occupation of the volume in the cell interior is about 7–40%^35^. It should be pointed out that the substrates used and the products formed are significantly smaller than both InhA and the crowding agents (except sucrose and glucose), suggesting that their presence should not influence the excluded volume during the progression of the reaction as substrates are converted into products. Interpretation of experimental results were based on definitions of apparent steady-state kinetic constants^16, 17^. Namely, the catalytic constant (*k*_cat_) represents a first-order rate constant that refers to the properties and reactions of the enzyme-substrate(s), enzyme-intermediate(s), and enzyme-product(s) complexes (including the chemical step, conformational changes and dissociation rate constants). The Michaelis-Menten constant (*K*_m_) gives an overall dissociation constant of all enzyme-bound species. The specificity constant (*k*_*cat*_/*K*_*m*_) gives an estimate for the apparent second-order rate constant either for DD-CoA binding to InhA:NADH binary complex (at fixed-saturating NADH concentration and varying DD-CoA concentrations) or NADH binding to InhA:DD-CoA binary complex (at fixed-saturating DD-CoA concentration and varying NADH concentrations). As linear reciprocal plots were observed for both substrates of InhA^9, 10^ and NADH and DD-CoA have been shown to bind to free enzyme^14, 36^, a rapid-equilibrium random-order mechanism appears to hold for InhA. Incidentally, it has been put forward that the *K*_m_ for each substrate in this type of mechanism represents the equilibrium dissociation constant for dissociation of the substrate from the ternary complex^37^.

The effects of crowding agents on *K*_m_ values may arise from changes in apparent equilibrium constant for an isomerisation process. The presence of a crowding agent may drive a protein conformational change to a more compact (closed) isomer. If substrate(s) can only bind to a more relaxed (open) protein isomer, there should be an increase in the overall dissociation constant value (*K*_m_) for the enzyme-catalyzed chemical reaction as the equilibrium was shifted to the closed isomer. The opposite holds if substrate(s) can only (or preferentially) bind to a more compact isomer. If interaction between enzyme and substrate leads to a more compact complex as compared to free enzyme, the presence of crowding agents can lead to a decrease in *K*_m_ value as the association process is favoured^3, 4^. For oligomeric proteins, molecular crowding agents may shift the equilibrium to higher order oligomeric forms to counterbalance the exclude volume^4^. If substrate can only (or preferentially) bind to the lower order oligomeric form of an enzyme, there will be an increase in *K*_m_ and a concomitant decrease in *k*_cat_ as velocity depends on the concentration of productive complexes or because a rate-limiting step involving product release became slower. When enzyme-substrate complex in the ground state is more compact than free species, a decrease in *K*_m_ may occur in the presence of crowding agents. However, it should be kept in mind that *K*_m_ gives an overall dissociation constant of all enzyme-bound species (including enzyme-product complex). The effects on *k*_*cat*_ are complex because, although crowding reduces diffusion, it increases thermodynamic activities of molecules. The overall rate of the reaction will be reduced if the rate of encounter of reactants is limited by diffusion^1^. In addition, there is a reduction in entropy in the presence of crowding agents that leads to an increase in the partial molar free energy (chemical potential) of the solute, which can be accounted for by the increase in the thermodynamic activity (or effective thermodynamic concentration) due to an increase in the activity coefficient value. Accordingly, enzyme-catalyzed chemical reactions may be enhanced in solutions containing crowding agents.

A reaction taking place in a solution containing macromolecules should have a standard free energy profile influenced by steric-repulsive interactions between the background particles and the reactants, the transition-state complex and/or the products^38^. During catalysis the transition-state may be expanded or contracted in crowded solutions and this could raise or lower the energy of activation, thereby affecting *V*_*max*_ (*k*_cat_)^3^. Hence, the effects of crowding agents on the thermodynamic activation parameters (*E*_*a*_, *∆H* ^*#*^, *∆S* ^*#*^, *∆G* ^*#*^) for InhA-catalyzed chemical reaction were assessed by measuring the dependence of *k*_*cat*_ on temperature (Fig. 3).

The experimental strategies were initially set out to evaluate whether or not there were crowding effects on InhA enzyme activity due to volume exclusion only. Surprisingly, the control experiment using sucrose showed a more convincing effect. Molecular dynamics efforts were thus sought to try to explain these unexpected results for sucrose. Moreover, the Arrhenius plot for PEG 6000 displayed a nonlinear pattern, which led to “soft” effects being invoked. As different effects were observed for the crowding agents here tested, it was deemed appropriate to interpret the results for the crowding agents in separate sections. A section on ficoll to evaluate the effect of the same polymeric chain with different sizes, and a section on dextran 70 to evaluate the effect of different crowding agents with similar sizes and chemically different polymeric chains are presented below. In addition, a separate section on the shift from hyperbolic to sigmoidal pattern for DD-CoA is presented.

### Hyperbolic Michaelis-Menten profile was shifted to sigmoidal one for DD-CoA

The presence of the crowding agents and controls (glucose and sucrose) altered from hyperbolic Michaelis-Menten to sigmoidal profile for variable DD-CoA substrate concentrations (Table ST1). The behavior of proteins in crowded milieu is related to the tendency of minimization of excluded volume either by changes of hydrodynamic volume of a protein or via changes in its oligomerization/association state^4^. Changes in the hydrodynamic volume are promoted by modulation of chemical equilibria that would alter the relative abundance of enzymes that exist in different conformations, shifting the equilibrium between these conformations^4, 39^. In the presence of crowding agents, there appears to be positive cooperativity for DD-CoA with values of approximately 2 for the cooperativity index, *n* (Table ST1). To the best of our knowledge, no allosteric sites have been reported for InhA, which suggests that there may exist an overall equilibrium between different conformational states of the enzyme in solution. To ascertain that the values were fitted to the correct equation, the residual values were plotted *versus* DD-CoA concentration for ficoll 70 (Figure SF4), showing that a sigmoidal profile more appropriately describes the experimental results. It has been shown positive cooperativity (*n* = 2) upon DD-CoA binding to free wild-type InhA, and a sequential or induced fit model with only one form of free InhA in solution was invoked to account for the results^36^. On the other hand, stopped-flow data on NADH binding to free isoniazid-resistant mutant InhA proteins suggested a mechanism with two forms of enzyme in equilibrium and only one of them being able to bind NADH^32^. The results here reported suggest that the crowding agents and controls (glucose and sucrose) may have shifted the equilibrium between two conformational states of free InhA in solution. As no sigmoidal profile was observed for NADH substrate (Table ST1), it is possible that only the interaction between DD-CoA and InhA is affected by this shift in isomerisation states of free enzyme. On the other hand, a sigmoidal kinetics without true cooperativity may be exhibited by a two-substrate enzyme that follows a random mechanism^17^. InhA is a two-substrate enzyme that follows rapid-equilibrium random-order mechanism, and a value of 0.57 μM (*K*_D_) for the hyperbolic binding of NADH to InhA^14^ and a value of 8.2 μM (*K*_*0.5*_) for the sigmoidal binding of DD-CoA to InhA^36^ were reported. The rate of InhA:DD-CoA → InhA:DD-CoA:NADH → products may be faster than InhA:NADH → InhA:NADH:DD-CoA → products reaction path for InhA. In this case, the dissociation constant value for InhA:NADH binary complex (*K*_D_ = 0.57 μM)^14^ should be larger than the dissociation constant for ternary complex upon NADH binding to InhA:DD-CoA (*K*_m_ = 113 μM). If [InhA] and [NADH] are held constant while [DD-CoA] is varied, at low concentrations of DD-CoA the enzyme will react mainly with NADH first and product formation will follow the slower path. As the concentration of DD-CoA increases, there will be a greater probability of DD-CoA binding to free InhA enzyme and the faster path of product formation will be followed. However, it does not appear to hold for InhA as a value of 0.57 μM for the dissociation constant for InhA:NADH binary complex formation has been determined by fluorescence spectroscopy^14^ and a value of 113 μM is here reported for NADH *K*_m_ (Table ST1), which represents the equilibrium dissociation constant for dissociation of NADH from InhA:DD-CoA:NADH ternay complex^37^. Notwithstanding, pre-steady-state kinetics of substrate binding to free InhA should be carried out in the presence of the crowding agents and controls or, for instance, sedimentation equilibrium analytical ultracentrifugation method could be employed in the presence of crowding agents and controls to ascertain whether or not there is an equilibrium between different oligomeric states of the enzyme.

### Effects of sucrose

The values of apparent steady-sate kinetic parameters for NADH and DD-CoA (Table ST1) substrates in Pipes 100 mM pH 7.0 are within experimental error to ones previously reported^9, 10, 14^. The values of *k*_cat_/*K*_m_ for InhA in the absence of crowding agents were 81 (±31) × 10^3^ M^−1^ s^−1^ and 120 (±23) 10^3^ M^−1^ s^−1^ for, respectively, NADH and DD-CoA (Table ST1). As the second-order rate constants for a diffusion-controlled enzymatic reaction should be in the 10^8^–1010 M^−1^ s^−1^ range^16, 17^, the *k*_cat_/*K*_m_ values suggest that InhA-catalyzed chemical reaction is not diffusion-limited.

Glucose (RH = 3.9 Å; Table ST1) was employed as negative control as it is a component unit of sucrose, ficoll and dextran oligomers. Sucrose (RH = 4 Å; Table ST1)^40^ a neutral osmolyte, was also chosen as control as it may be regarded as a mimic of the monomeric unit of ficoll polysacharides. Although there appears to be a decrease in *k*_*cat*_/*K*_m_ value for NADH at 200 mg mL^−1^ glucose concentration (Fig. 1, Table ST1), no systematic effect could be clearly observed. No statistically significant effects were observed for the steady-state kinetic parameters for either DD-CoA or NADH substrates in solutions containing glucose (Fig. 2, Tables ST1 and ST2). The *K*_*m*_ and *k*_*cat*_ values for NADH increased up to 100 mg mL^−1^ and decreased at 200 mg mL^−1^ sucrose concentration (Fig. 1). The *k*_*cat*_/*K*_*m*_ values decreased as a function of increasing sucrose concentration (Fig. 1C, Table ST1). The *K*_*0.5*_ and *k*_*cat*_ values for DD-CoA decreased in a concentration-dependent manner (Fig. 2, and Tables ST1 and ST2). Significant correlations between increasing concentrations of sucrose and decreasing *k*_*cat*_ and *K*_0.5_ values for DD-CoA were observed (Table ST2). As InhA follows a rapid-equilibrium random-order mechanism^9, 10, 14, 36^, the decreasing *k*_*cat*_/*K*_*m*_ values for NADH (Table ST1) in sucrose solution represents a solid piece of experimental evidence (even though not statistically significant). Moreover, the MD simulation results lend support to the effects of sucrose on InhA enzyme. Sugars, such as sucrose, have been described to alter the viscosity, polarity, and dielectric constant of the solution, to affect water activity, and to stabilize native conformation of globular proteins^41, 42^. The effects on InhA steady-state kinetic parameters cannot be attributed to increasing viscosity of sucrose solutions as their values are lower than the ones of the other crowding agents (Table ST1). Sucrose, ficoll 70, and dextran 70 solutions at the same concentrations have similar polarity values^34^. Accordingly, polarity likely plays no role in sucrose solutions as no effects on steady-state kinetic parameters were observed for ficoll 70 and dextran 70 (Figs 1 and 2) as will be subsequently discussed. It has been reported that there occurs a quench in intrinsic protein fluorescence upon NADH^14^ and DD-CoA^36^ binding to free InhA. To evaluate whether the effects on steady-state kinetics parameters observed for sucrose were due to InhA:sucrose binary complex formation, intrinsic protein fluorescence measurements of sucrose binding to InhA were carried out. No sucrose binding to free InhA could be detected by fluorescence spectroscopy measurements (Figure SF5, panel D). However, these data cannot rule out sucrose binding to InhA resulting in no change in intrinsic protein fluorescence and/or sucrose binding to an allosteric site. Attempts to carry out isothermal titration calorimetry measurements of sucrose binding to InhA were to no avail as the viscosity of sucrose stock solution impeded collection of reliable data. As no systematic effects were observed for glucose (Fig. 1, Table ST2), the effects on InhA steady-state kinetic parameters appear to be specific to sucrose. In agreement with this proposal, the MD simulation results suggest that InhA exhibits a more compact conformation in sucrose as compared to glucose solution, and that the latter is more similar to PIPES buffer solution (Table ST4).

The larger *E*_*a*_ and *∆H* ^*#*^ values suggest a less favourable activation process for InhA-catalyzed chemical reaction in solution containing sucrose as compared to buffer, whereas the larger *∆S* ^*#*^ value suggests a more favourable process (Table 1). At any rate, the *∆G* ^*#*^ of activation for sucrose solution is comparable to buffer (Table 1).

Results of molecular dynamics simulations of InhA in sucrose solutions suggest that the decreasing values of apparent second-order rate constants (*k*_*cat*_/*K*_*m*_) for NADH as a function of increasing sucrose concentrations (Fig. 1) may be accounted for by the more compact conformational states adopted by InhA subunits (Fig. 5). The latter enzyme conformers would result in a decrease in effective collisions between InhA and substrate(s), and ensuing reduction in chemical conversion of substrates into products and/or release of the latter into bulk solution. The more positive value of enthalpy of activation (*∆H* ^*#*^) in the presence of sucrose at 200 mg mL^−1^ as compared to buffer (Table 1) suggests reduction in redistribution of the network of interatomic interactions (hydrogen bonds, van der Waals, etc) between the reacting species including the solvent^43^. On the other hand, there appears to be an increase in the relative degrees of disorder in sucrose solution as compared to buffer, which is borne out by the larger entropy of activation (*∆S* ^*#*^, Table 1). It is tempting to suggest that the release of “bound” water molecules resulting from a more compact conformational state of InhA (Fig. 5) may account for by the increased *∆S* ^*#*^ value in sucrose solutions.

### Effects of PEG 6000

An estimate for the hydrodynamic radius (Eq. S3) of polymer in the solution of reaction was obtained from the intrinsic viscosity value (Eq. S4) for the reaction mixture. In the solution of PEG 6000, the smallest polymeric crowding agent used (RH = 21.2 Å; Table ST1) whose hydrodynamic radius is similar to InhA (28.8 Å), a complex effect on the apparent steady-state kinetic parameters for NADH (Fig. 1) and DD-CoA (Fig. 2) were observed. The *K*_*m*_ and *k*_*cat*_ values for NADH were larger than the control up to 100 mg mL^−1^ and decreased at 200 mg mL^−1^ (Table ST1). Notwithstanding, the *k*_cat_/*K*_m_ for NADH remained fairly constant (Table ST1). For DD-CoA substrate, the kinetic parameters were not changed, except a lower *k*_*cat*_ value was observed at 200 mg mL^−1^ (Table ST1). Using PEG as crowding agent, larger *K*_*m*_ and lower *k*_*cat*_ values were observed for horseradish peroxidase^44^, and decreased enzyme activity in a concentration-dependent manner was measured for urease^45^. Non-linear effect in the presence of PEG 8000 was observed for *K*_m_ and *k*_cat_ values of *Plasmodium falciparum* purine nucleoside phosporylase (PfPNP), whereas *k*_cat_/*K*_m_ values decreased as a function of polymer concentration^46^. These authors showed by fluorescence spectroscopy binding of crowding agents to the active site loop of this hexameric enzyme^46^. A proposal has been put forward in which the more rigid active site loop environment of PfPNP would account for the increase of *K*_m_ values at low concentrations of crowding agents whereas at larger concentrations of the latter an increase in substrates and enzyme concentrations would lower the *K*_m_ values^46^. A decrease in active site loop flexibility of PfPNP and concomitant reduction in product release has been invoked to explain the decrease in *k*_cat_ values in the presence of PEG 3300^46^. The large viscosity value for the solution containing PEG 6000 at 200 mg mL^−1^ (Table ST1) cannot be invoked to explain the lower *k*_cat_ values for NADH (Table ST1) and DD-CoA (Table ST1), as this value is somewhat similar to ficoll 400 at 200 mg mL^−1^ (Table ST1). In agreement with this proposal, no effect on the apparent second-order rate constant for NADH (Fig. 1, Table ST1) and modest effects on *K*_0.5_, *n* and *k*_*cat*_ for DD-CoA (Fig. 2, Table ST1) were observed for PEG 6000, even though this crowding agent at 200 mg mL^−1^ displayed the largest fractional volume occupancy (Table ST1). Although somewhat speculative, the increase in *K*_*m*_ and *k*_*cat*_ values for NADH up to 100 mg mL^−1^ of PEG 6000 may be ascribed to a shift in equilibrium to a more compact isomeric form of InhA in solution (increasing *K*_*m*_ values) with concomitant increase in the chemical potential of solutes (increasing *k*_*cat*_ values). The opposite effects observed for PEG 6000 at 200 mg mL^−1^ may be due to different step(s) becoming rate limiting slowing down the enzyme-catalyzed chemical reaction and protein equilibrium shifting to an isomeric form with increased affinity for the substrate(s). At any rate, whether or not there may be a shift in an equilibrium between different oligomeric states of InhA in solutions containing PEG 6000 or isomerisation to a more compact form, studies of, respectively, sedimentation equilibrium analytical ultracentrifugation and coarse grain molecular dynamics have to be pursued. In addition, as discussed in the next paragraph, “soft” interactions appear to play a role in solutions containing PEG 6000 at 200 mg mL^−1^, which may, for instance, decrease substrate(s) off-rate constant(s) to yield free enzyme (decreasing *K*_*m*_) and reduce the rate constant of a step linked to substrates conversion to products (decreasing *k*_*cat*_). Moreover, whether or not PEG 6000 can bind to free InhA and/or slow down product release as proposed for PfPNP^46^, both binary complex formation and pre-steady-state kinetics studies ought to be pursued.

A nonlinear pattern in Arrhenius plot was observed for InhA solution containing PEG 6000 (Fig. 3), whereas a linear pattern was observed for a solution of this crowding agent in the absence of enzyme (Table ST2, Figure SF3). Accordingly, the nonlinearity of the Arrhenius plot for PEG 6000 is a property of InhA-catalyzed chemical reaction in this solution. The two sets of thermodynamic parameters (Table 1) suggest that lower temperatures yielded larger *E*_*a*_, *∆H* ^*#*^ and *∆S* ^*#*^ values, whereas the *∆G* ^*#*^ values were fairly similar (Table 1). This effect may suggest that either different steps became rate-limiting at different temperatures or that the change in heat capacity at constant pressure (*∆C*_*p*_^*#*^) is different from zero^16, 47^. The thermodynamic signatures of macromolecular crowding on protein conformation or kinetics are commonly related to minimization of excluded volume which is mainly described in terms of entropic contributions^4^. The entropy of activation is related to the facility of formation of the activated enzyme:substrate(s) complex. This complex could be more easily formed if the degree of order is lower than the enzyme:substrate(s) in the ground state (*∆S* ^*#*^ > 0), or less easily formed if the degree of order is higher than the complex in the ground state (*∆S* ^*#*^ < 0)^48^. The entropy values for PEG 6000 (and for all other crowding agents) were negative suggesting a higher degree of order for the enzyme:substrate(s) activated complex as compared to enzyme:substrate(s) in the ground state. Owing to the nonlinear Arrhenius plot for PEG 6000, the activation entropy *∆S* ^*#*^ value was found more favourable (even though negative) at lower temperatures than at larger ones (Table 1). The entropy change mainly reflects two contributions: changes in solvation entropy and changes in conformational entropy^43^. Upon substrate binding en route to chemical catalysis, desolvation may occur, water is released and a gain in solvent entropy is observed. This gain is particularly important for hydrophobic groups. At the same time, the ligand and certain groups in the protein lose conformational freedom upon complex formation and likely during catalysis, resulting in a negative change in conformational entropy. The enthalpy primarily reflects the strength of the interactions of the ligand with the protein and activated enzyme:substrate(s) complex (e.g. van der Waals, hydrogen bonds, etc.) relative to those existing with the solvent either in the presence or absence of crowding agents. Large positive activation enthalpy (*∆H* ^*#*^) values are typical of unfavorable processes. The larger *∆H* ^*#*^ value at low temperatures for PEG 6000 as compared to higher temperatures suggests more favourable macromolecular interactions at larger temperatures (Table 1). The free activation energy *∆G* ^*#*^ can represent the energy barrier required to initiate a reaction and it is expected to be lowered due to the excluded-volume effect^38^. It can also be regarded as the variation of the Gibbs energy between the activated enzyme:substrate(s) complex and enzyme:substrate(s) in the ground state^48^. Even though the nonlinear Arrhenius plot for PEG 6000 showed different *∆S* ^*#*^ and *∆H* ^*#*^ values (Table 1), there was no change in *∆G* ^*#*^ in the temperature range tested. In addition, the *∆G* ^*#*^ values for the crowding agents were similar to buffer alone (Table 1), suggesting that excluded volume effects did not facilitate stable activated *ES* ^*#*^ complex formation. Incidentally, enthalpy-entropy compensation resulting in temperature-independent *∆G* ^*#*^ values for protein:ligand interactions are fairly common^49^. It has been pointed out that macromolecular crowding effects (or depletion force) have been predicted by the Asakura and Oosawa model to be entirely entropic as it relies on fully steric interactions that are temperature independent^50^. Nonlinearity of Arrhenius plots suggest that “soft” interactions (attractive or repulsive) may play a role in macromolecular crowding effects. Accordingly, the results for PEG 6000 (Fig. 3) suggest that there may be an enthalpic component that contributes to its effect on InhA enzyme activity. In agreement with this proposal, it has been shown that chemical interactions between high molecular weight PEGs and proteins occur concomitantly to excluded volume effects^51^.

### Effects of ficoll 70 and ficoll 400

As pointed out above, a useful criterion to discriminate between non-specific crowding effects and specific macromolecular interactions is to collect data at the same weight concentration for large and small crowding agents having the same polymeric chain, in which the latter should cause greater effects than the former^33^. The hydrodynamic radius for ficoll 70 was 34.5 Å and for ficoll 400 was 69.1 Å (Table ST1). These results are in reasonably good agreement with the values listed by Kuznetsova *et al*.^4^. As the hydrodynamic radius of ficoll 70 is somewhat similar to tetrameric InhA (28.8 Å), the proposal that effective conditions for studying macromolecular crowding effects should include the test molecule and crowding agent having similar sizes has been satisfied^4^.

The presence of ficoll 70 and ficoll 400 had no effect on the apparent kinetic parameters for NADH substrate (*K*_*m*_, *k*_*cat*_ and *k*_*cat*_/*K*_*m*_) when compared with diluted buffer (Fig. 1). Moreover, no noticeable effect could be observed for ficoll 70 and ficoll 400 using DD-CoA as variable substrate (Fig. 2B, Table ST1). The fraction of volume occupied and density for these crowding agents were fairly similar at the same concentration values at 25 °C (Table ST1). In addition, increasing solution viscosity has no effect on enzyme reaction (Table ST1), which is in agreement with the InhA rate of catalysis not being limited by diffusion. These results suggest that there are no macromolecular crowding effects on the InhA steady-state kinetics parameters for NADH and DD-CoA substrates.

The linear Arrhenius plot for ficoll 70 and ficoll 400 (Fig. 3) suggests that there is no change in the rate-limiting step over the temperature range employed in the studies here presented. The *E*_*a*_, *∆H* ^*#*^, *∆S* ^*#*^, and *∆G* ^*#*^ values for Pipes 100 mM, ficoll 70 and ficoll 400 solutions were within experimental error (Table 1). These results are in agreement with the steady-state kinetic results.

### Effects of dextran 70A

value of 58.8 Å for the hydrodynamic radius of dextran 70 (Table ST1) was estimated, which is in agreement with the value of 58 Å reported in the Amersham Biosciences brochure by A. N. de Belder.

The effects of dextran 70 on the apparent steady-state kinetics constants were modest for NADH (Fig. 1) and DD-CoA (Fig. 2) substrates, and not statistically significant (Table ST2). The values for the fractional volume occupancy of dextran 70 were comparable to both ficoll 70 and 400 at the same mass concentrations (Table ST1). As one criterion to discriminate between crowding effects and specific interactions is to observe similar effects with chemically different crowding agents having similar radii, the results for ficoll 400 (RH = 69.1 Å; Table ST1) and dextran 70 (RH = 58.8 Å; Table ST1) suggest that there is no crowding effect of dextran 70 on InhA kinetic parameters. Although viscosity values were larger for dextran 70 as compared to ficoll, the modest effects of the former on the kinetic parameters are consistent with InhA-catalyzed chemical reaction not being diffusion-limited. However, it should be pointed out that enzyme activity measurements for dextran 70 at 200 mg ml^−1^ were not included in this study as no reliable data could be obtained, even using a stopped-flow apparatus for rapid mixing. Changes in absorbance measurements were observed in the absence of enzyme (data not shown), suggesting possible physical or chemical effects in solutions containing dextran 70 at 200 mg mL^−1^. Since the neutral and relatively hydrophilic nature of dextran 70 should minimize specific interactions with proteins, no specific chemical interactions are expected between this crowding agent and reactant molecules^4, 52^. Laminar flow and different refraction indices may account for these unexpected observations at large dextran 70 concentrations^52, 53^.

## Conclusions

As the intracellular milieu is highly crowded, the effects of *in vitro* crowding on biochemical reactions should be pursued to try to mimic *in vivo* conditions. However, as the intracellular components are heterogeneous with respect to shape, size and possible macromolecular chemical interactions, *in vitro* experiments will never perfectly mimic the physiological *in vivo* milieu. It has been proposed that quinary structure should be implemented into models to study protein function in the crowded cellular environment^54^, and that polymer-induced changes in the solvent properties of aqueous media should be taken into account^55^. As “soft” interactions were invoked to explain the results for PEG 6000 and only sucrose showed a decrease in both *k*_cat_/*K*_m_ for NADH and *k*_cat_ for DD-CoA, the results presented in our manuscript do not unequivocally meet the criteria for crowding effect due to exclude volume only. Incidentally, it has been proposed that large molecules are less effective at crowding than water and ions (the smaller the better)^56^. Accordingly, further efforts are worth pursuing to try to evaluate macromolecular crowding effects, if any, on InhA enzyme activity, such as concentrated solutions of various synthetic and biological polymers^4^, effects of osmolytes combined with macromolecular crowding^57^, increasing sizes of the same polymer^58^. These efforts aim at developing an *in vitro* model to assay InhA activity that would serve as a surrogate of *in vivo* intracellular medium for anti-tuberculosis drug screening campaigns.

## Electronic supplementary material

Supplementary Information
