# Supplementary material for: Observed crowding effects on Mycobacterium tuberculosis 2-trans-enoyl-ACP (CoA) reductase enzyme activity are not due to excluded volume only
Source: Sci Rep. 2017 Jul 28;7:6826. doi: 10.1038/s41598-017-07266-w (PMC5533716; doi:10.1038/s41598-017-07266-w)
Supplement: Supplementary file 1 — Supplementary Information [file 41598_2017_7266_MOESM1_ESM.pdf]

SUPPLEMENTARY INFORMATION:

OBSERVED CROWDING EFFECTS ON *MYCOBACTERIUM TUBERCULOSIS* 2-*TRANS*-ENOYL-ACP (COA) REDUCTASE ENZYME ACTIVITY ARE NOT DUE TO EXCLUDED VOLUME ONLY.

Mariane Rotta[†‡], Luis F. S. M. Timmers[§¥], Carlos Sequeiros-Borja[§¥], Cristiano V. Bizarro[†], Osmar N. de Souza[§¥], Diogenes S. Santos[†*], Luiz A. Basso[†‡*]

[†]Instituto Nacional de Ciência e Tecnologia em Tuberculose (INCT-TB), Centro de Pesquisas em Biologia Molecular e Funcional (CPBMF), Pontifícia Universidade Católica do Rio Grande do Sul (PUCRS), Porto Alegre, RS, Brazil.

[‡]Programa de Pós-Graduação em Medicina e Ciências da Saúde, PUCRS, Porto Alegre, RS, Brazil.

[§]Laboratório de Bioinformática, Modelagem e Simulação de Biossistemas (LABIO), Faculdade de Informática, PUCRS, Porto Alegre, RS, Brazil

[¥]Laboratório de FarmInformática (FarmInf), Faculdade de Farmácia, PUCRS, Porto Alegre, RS, Brazil.

Contents:

1. Assay of enzyme activity for dextran 70 at 200 mg mL^{-1}

Table ST1. Apparent steady-state kinetics parameters for NADH and DD-CoA substrates, and physical chemical properties of crowding agents and their aqueous solutions.

Table ST2 Statistical analysis of effects of different molecular crowding agents on the apparent steady-state kinetic parameters of InhA.

Figure SF1. Plots of density *versus* concentrations of crowding agents.

2. Calculation of hydrodynamic radius

Figure SF2. Non-linear function of reduced specific viscosity (η_{sp}/c_m in g cm^{-3}) on mass concentration of solute in the solution (c_m in g cm^{-3}).

Table ST3. Variation of densities and viscosities of the crowding agents with temperature

Figure SF3. Arrhenius plot for the crowding agents (temperature dependence of $ln\ \eta$).

Figure SF4. Plot of velocity as a function of increasing DD-CoA concentration in the presence of ficoll 70 at 200 mg mL^{-1}.

Figure SF5. Plot of florescence spectroscopy data for sucrose.

Figure SF6. B-factor for all MD simulations of InhA during the production phase.

Figure SF7. Distribution of the volume of InhA active site cavity.

Figure SF8. Box plot for the variance of the volume of the volume of InhA active site cavity during the simulation at 200 mg mL^{-1} sucrose concentration.

Figure SF9. Distribution of active site cavity of InhA in the absence (blue) and in the presence of 25 mg ml^{-1} (red) and 200 mg ml^{-1} (green) of sucrose.

Table ST4. Games-Howell (GH) test using a confidence level of 95%.

1. Assay of enzyme activity for dextran 70 at 200 mg mL^{-1}

Mixing difficulties were observed in the enzyme activity measurements for dextran 70 at 200 mg ml^{-1} in the continuous spectrophotometric assay. Therefore an Applied Photophysics SX.18MV-R stopped-flow spectrofluorimeter on absorbance mode, operated at 25 °C, using the 10 mm light path, was employed in attempts to overlap this problem. All measurements were monitored for 60 s at 340 nm using pipes 100 mM (pH 7.0) as described in the experimental section. Prior to conducting the steady-state kinetic determinations, three control experiments were performed to guarantee reliable data. First, a solution of NADH, DD-CoA, and dextran 70 was prepared in pipes 100 mM pH 7.0 and loaded into one syringe and mixed with a solution containing dextran 70 and pipes 100 mM pH 7.0 in the second syringe. After mixing, the concentration of NADH, DD-CoA, and dextran were 200 μM, 105 μM, and 200 mg mL^{-1}, respectively. Second, in order to evaluate possible interactions between the substrates and the crowding agent, a solution of NADH or DD-CoA, and dextran 70 was prepared in pipes 100 mM pH 7.0 and loaded into one syringe and mixed with a solution containing dextran 70 and pipes 100 mM pH 7.0 in the second syringe. The final concentrations were retained as described above. Finally, to study effects of the crowding agent dilution in the measurements two solutions of dextran 70 (200 mg mL^{-1}) prepared in pipes 100 mM pH 7.0 were loaded into each of the syringes and mixed. For all of these controls eight mixing events were collected with 400 data points recorded for each and the average of five different reading was analyzed.

Table ST1 Apparent steady-state kinetics parameters for NADH and DD-CoA substrates, and physical chemical properties of crowding agents and their aqueous solutions.

		NADH			DD-CoA									
	Concentration (mg mL^{-1})	K_m (μM)	k_{cat} (s^{-1})	k_{cat}/K_m x 10^3 (M^{-1} s^{-1})	$K_m/K_{0.5}$ (μM)	n	k_{cat} (s^{-1})	Density (g cm^{-3})	Viscosity (mPa s)	Specific Viscosity	RH* (Å)[a]	Intrinsic RH** (Å)[b]	Volume fractional (%)	Partial specific volume (mL g^{-1})
Pipes 100 mM	-	113 ± 39	9 ± 1	81 ± 31	57 ± 11	-	6.8 ± 0.6	1.03 ± 0.02	1.14965 ± 0.0	-	-	-	-	-
Ficoll 70	25	128 ± 16	9.1 ± 0.5	71 ± 9	30 ± 5	2.0 ± 0.6	5.7 ± 0.6	1.0196 ± 0.0	1.2438 ± 0.0004	0.082	33.14	34.5	1.65	0.6
	50	159 ± 16	10.0 ± 0.5	63 ± 4	35 ± 5	2.1 ± 0.6	5.8 ± 0.5	1.0257 ±0.0	1.5209 ± 0.0002	0.323	41.54		3.3	
	100	127 ± 13	8.6 ± 0.3	67 ± 7	34 ± 4	2.0 ± 0.5	5.9 ± 0.5	1.0437 ± 0.0	2.246 ± 0.002	0.954	47.30		6.6	
	200	112 ± 15	9.1 ± 0.5	81 ± 12	34.0 ± 0.5	1.9 ± 0.4	6.2 ± 0.6	1.0766 ± 0.0	8.23 ± 0.09	6.159	69.91		13.2	
Ficoll 400	25	127 ± 18	10.0 ± 0.6	79 ± 12	33 ± 3	2.2 ± 0.5	6.0 ± 0.4	1.0186 ± 0.0	1.36 ± 0.0	0.183	77.43	69.1	1.5	0.68
	50	152 ± 22	11.0 ± 0.6	72 ± 11	35 ± 4	2.2 ± 0.5	6.3 ± 0.4	1.0257 ± 0.0	1.907 ± 0.0	0.659	89.63		2.98	
	100	121 ± 22	10 ± 2	87 ± 22	37 ± 5	2.2 ± 0.5	6.5 ± 0.5	1.0388 ± 0.0	3.7154 ± 0.0001	2.231	112.27		5.96	
	200	142 ± 68	9 ± 2	61 ± 33	39.8 ± 0.8	1.9 ± 0.7	4.9 ± 0.7	1.0718 ± 0.0002	14.46 ± 0.01	11.578	154.27		11.92	
Dextran 70	25	154 ± 21	11.0 ± 0.6	71 ± 10	27 ± 4	2.4 ± 0.7	6.1 ± 0.5	1.021 (± 0.0)	1.844 (± 0.001)	0.604	64.54	58.8	1.41	0.56
	50	165 ± 17	13.2 ± 0.6	80 ± 9	41 ± 5	1.8 ± 0.3	7.8 ± 0.6	1.033 (± 0.0)	3.337 (± 0.007)	1.903	75.07		2.87	
	100	103 ± 19	10.6 ± 0.7	103 ± 20	42 ± 5	2.7 ± 0.9	7.3 ± 0.6	1.055 (± 0.0)	8.90 (± 0.02)	6.741	90.83		5.67	
	200	-	-	-	-	-	-	1.096 (± 0.0)	42.04 (± 0.02)	35.550	125.48		11.34	
PEG 6000	25	173 ± 26	12.2 ± 0.7	70 ± 11	31 ± 5	2.4 ± 0.9	6.4 ± 0.6	1.016 (± 0.0)	1.572 (± 0.007)	0.367	24.08	21.2	1.97	0.79
	50	187 ± 26	12.7 ± 0.8	68 ± 11	32 ± 5	2.4 ± 0.8	6.6 ± 0.6	1.022 (± 0.0)	2.450 (± 0.001)	1.131	27.81		3.9	
	100	190 ± 38	12 ± 1	64 ± 14	31 ± 5	2.1 ± 0.7	6.8 ± 0.7	1.031 (± 0.0)	4.97 (± 0.01)	3.323	31.62		7.9	
	200	64 ± 13	5.0 ± 0.3	78 ± 17	32 ± 12	1.7 ± 0.8	3.9 ± 0.9	1.051 (± 0.0)	15.80 (0.09)	12.743	39.30		15.8	
Sucrose	25	199 ± 12	11.6 ± 0.3	58 ± 4	32 ± 4	2.1 ± 0.4	6.5 ± 0.5	1.023 (± 0.0)	1.068 (± 0.004)	-	-	4[c]	1.35	0.53
	50	259 ± 38	12 ± 1	46 ± 8	29 ± 4	2.2 ± 0.6	5.9 ± 0.6	1.034 (± 0.0)	1.167 (± 0.0)	0.015	2.5		2.65	
	100	263 ± 18	11.1 ± 0.4	42 ± 3	24 ± 3	2.5 ± 0.6	5.1 ± 0.4	1.0578 (± 0.0)	1.414 (± 0.0)	0.230	5		5.3	
	200	150 ± 31	5.8 ± 0.5	39 ± 9	19 ± 4	2.0 ± 0.8	3.9 ± 0.5	1.104 (± 0.0)	2.189 (± 0.003)	0.904	6.2		10.6	
Glucose	25	82 ± 13	8.1 ± 0.5	99 ± 8	73 ± 28	1.7 ± 0.4	9.5 ± 3	1.0142 (± 0.0)	1.0043 (± 0.0)	-	-	3.9[d]	1.5	0.6
	50	81 ± 15	8.6 ± 0.5	106 ± 20	73 ± 28	1.6 ± 0.3	10 ± 3	1.0257 (± 0.0)	1.0920 (± 0.0)	-	-		3	
	100	126 ± 26	9.5 ± 1	75 ± 29	49 ± 5	2.0 ± 0.2	6.8 ± 0.4	1.0410 (± 0.0)	1.2591 (± 0.0)	0.095	3		6	
	200	187 ± 25	9 ± 0.5	49 ± 7	36 ± 3	1.9 ± 0.2	5.0 ± 0.3	1.0850 (± 0.0)	1.8277 (± 0.0)	0.6	4.4		12	

* Hydrodynamic Radius; ** Intrinsic hydrodynamic radius; [a]Calculated from equation (S2); [b]Calculated from equation (S3); [c]Graziano, G. How does sucrose stabilize the native state of globular proteins? *Int. J. Biol. Macromol.* **2012**, 50, 230–235; [d]Sabek, O. M. *et. al.*, Characterization of a nanogland for the autotransplantation of human pancreatic islets. *Lab Chip*, **2013**, 13, 3675-3688.

Table ST2 Statistical analysis of effects of different molecular crowding agents on the apparent steady-state kinetic parameters of InhA.

Crowding agent	NADH						DD-CoA			
	K_m		k_{cat}		k_{cat}/K_m		$K_{0.5}$		k_{cat}	
	r	P-value	r	P-value	r	P-value	r	P-value	r	P-value
Ficoll 70	-0.279	0.650	-0.170	0.785	0.232	0.707	0.4492	0.5508	-0.1247	0.8417
Ficoll 400	0.430	0.470	-0.283	0.644	-0.644	0.241	0.9793	0.0207	-0.8323	0.0804
Dextran 70	-0.259	0.741	0.355	0.645	0.815	0.185	0.7936	0.4164	0.5110	0.4890
PEG 6000	-0.519	0.370	-0.661	0.224	0.033	0.957	0.4663	0.5337	-0.8600	0.0615
Sucrose	0.020	0.974	-0.681	0.206	-0.775	0.124	-0.9797	0.0203	-0.9936	**0.0006**
Glucose	0.870	0.055	0.409	0.495	-0.802	0.103	-0.9558	0.0442	-0.6670	0.2188

A Pearson correlation analysis was performed to detect linear correlations between apparent steady-state kinetic parameters (K_m or $K_{0.5}$, k_{cat}, k_{cat}/K_m) of InhA and different concentrations of crowding agents. The statistical significance of both decreasing (negative values) and increasing (positive values) Pearson correlation coefficients (**r**) was evaluated using a two-tailed test. P-values < 0.01 are highlighted in **bold**.

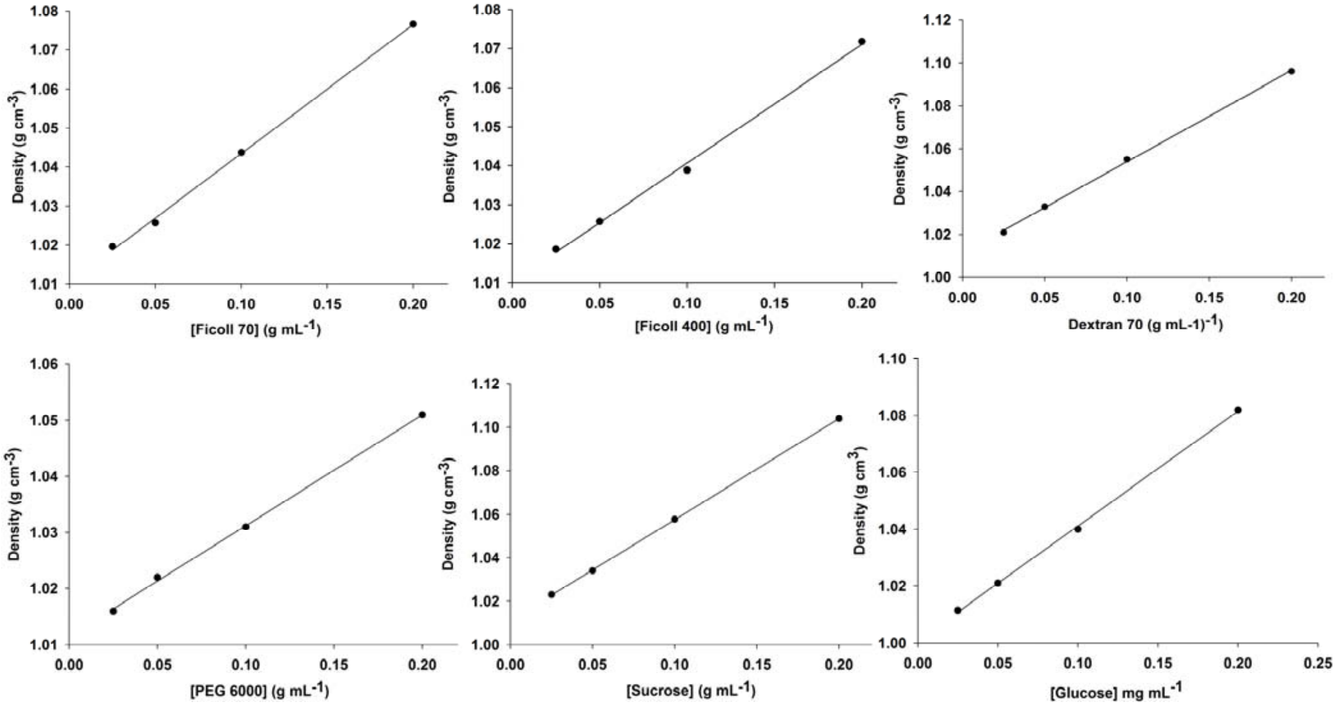

Supplementary Figure SF1. Plots of density *versus* concentrations of crowding agents. The linear correlation shows that there is no concentration dependence of the partial specific volumes.[1]

2. Calculation of hydrodynamic radius

To provide an estimate of the hydrodynamic radius of crowding agents (given in Table ST1), the specific viscosity was determined by the following equation S1:[2]

$$\eta_{sp} = \frac{\eta - \eta_0}{\eta_0} \quad \text{(S1)}$$

Where η and η_0 are, respectively, fluid viscosity of a solution containing crowding agent and buffer (Pipes 100 mM pH 7.0) only. The terms η and η_0 are dimentionless and positive. For a solution containing *N* spherical solute molecules, equation S2 describes the relationship between η_{sp} and the radius *R*:

$$\eta_{sp} = \frac{10\pi R^3 c_m}{3m} \quad \text{(S2)}$$

In which c_m is the mass concentration of the solute in solution (g mL^{-1} or g cm^{-3}), and *m* is the molecular mass of the solute (g $molecule^{-1}$). The specific viscosity (η_{sp}) is a colligative property as it depends on polymer concentration. The intrinsic viscosity ([η], in cm^3 g^{-1}) is given by the following relationship:

$$[\eta] \equiv \lim_{c_m \to 0}\left(\frac{\eta_{sp}}{c_m}\right) = \frac{10\pi R^3}{3m} \quad \text{(S3)}$$

The limit of infinite dilution ($c_m \to 0$) is required to define a viscosity property that is intrinsic to the solute (concentration independent) in a given solution. This limiting condition is needed to define the intrinsic viscosity ([η] in cm^3 g^{-1}) as the reduced

specific viscosity (η_{sp}/c_m in cm^3 g^{-1}) is polymer concentration dependent due to shear forces between the dissolved macromolecule and the solvent medium. The latter can be described by equation 4:

$$\frac{\eta_{sp}}{c_m} = [\eta] + k[\eta]^2 c_m + k' c_m^2 \qquad \text{(S4)}$$

Where k is the Huggins constant. The intrinsic viscosity ([η], in cm^3 g^{-1}) is an intrinsic property of the solute ($10\pi R^3/3m$), and thereby does not depend on the amount of solute present in solution.

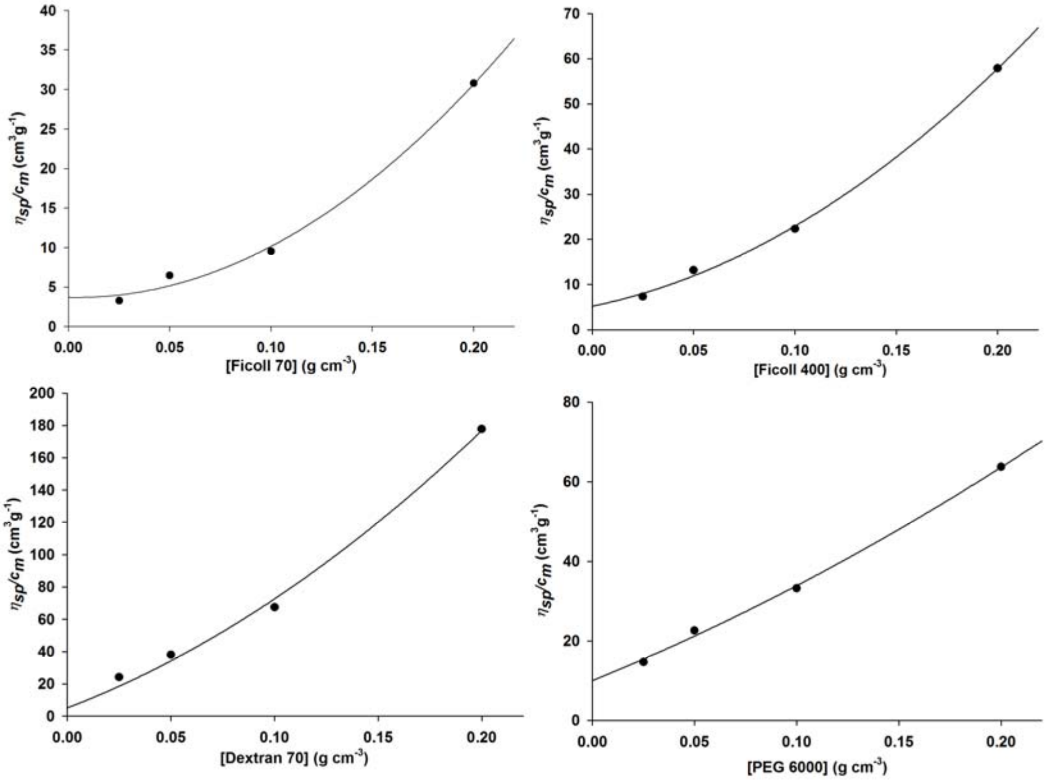

Supplementary Figure SF2. Non-linear function of reduced specific viscosity (η_{sp}/c_m in g cm^{-3}) on mass concentration of solute in the solution (η_{sp}/c_m in cm^3 g^{-1}). The data were fitted to Equation S4, yielding values for the intrinsic viscosity ([η] in cm^3 g^{-1}).

Table ST3. Variation of densities and viscosities of the crowding agents with temperature

Crowding agent[a]	Temperature (°C)	Viscosity (mPa s)	Density (g cm^{-3})
Pipes 100 mM	15	1.44725 (±0.017)	1.0380 (± 0.0)
	20	1.27485 (± 0.00125)	1.03695 (± 0.0015)
	25	1.13265 (±0.00145)	1.03545 (± 0.0)
	30	1.01275 (± 0.0)	1.03365 (± 0.0015)
	35	0.91375 (± 0.0007)	1.03155 (± 0.0)
	40	0.82773 (± 0.00036)	1.02945 (± 0.0)
Ficoll 70	15	11.371 (± 0.006)	1.07965 (± 0.0)
	20	9.68535 (± 0.009)	1.0783 (± 0.0002)
	25	8.1360 (± 0.0015)	1.0766 (± 0.0)
	30	7.22095 (± 0.0008)	1.07465 (± 0.00015)
	35	6.31995 (± 0.00135)	1.07225 (± 0.00015)
	40	5.57995 (± 0.0073)	1.06995 (± 0.00015)
Ficoll 400	15	19.8715 (± 0.023)	1.07535 (± 0.00015)
	20	16.859 (± 0.002)	1.07345 (± 0.00015)
	25	14.4605 (± 0.0155)	1.0718 (± 0.0002)
	30	12.494 (± 0.002)	1.06995 (± 0.00015)
	35	10.8995 (± 0.0025)	1.06795 (± 0.00015)
	40	9.58265 (± 0.00145)	1.0658 (± 0.0002)
PEG 6000	15	20.892 (± 0.0018)	1.05625 (± 0.00025)
	20	17.5625 (± 0.0015)	1.0544 (± 0.0001)
	25	14.9475 (± 0.00175)	1.05225 (± 0.00025)
	30	12.832 (± 0.003)	1.04975 (± 0.00015)
	35	11.1295 (± 0.0025)	1.0473 (± 0.0001)
	40	9.73455 (± 0.0)	1.04475 (± 0.00265)
Sucrose	15	2.9399 (± 0.0033)	1.10865 (± 0.00015)
	20	2.53275 (± 0.00245)	1.10715 (± 0.00015)
	25	2.198 (± 0.0006)	1.10525 (± 0.0)
	30	1.9319 (± 0.003)	1.10295 (± 0.0)
	35	1.70935 (± 0.00255)	1.10075 (± 0.0)
	40	1.5242 (± 0.002)	1.0985 (± 0.0)
Glucose	15	2.4620 (± 0.0)	1.0839 (± 0.0001)
	20	2.0548 (± 0.0001)	1.0840 (± 0.00015)
	25	1.8277 (± 0.0)	1.0850 (± 0.0001)
	30	1.5959 (± 0.0001)	1.0833 (± 0.0)
	35	1.3866(± 0.0)	1.0788 (± 0.0)
	40	1.2363(± 0.0)	1.0787 (± 0.0)

[a]The solutions of crowding agents are at a concentration of 200 mg mL^{-1}.

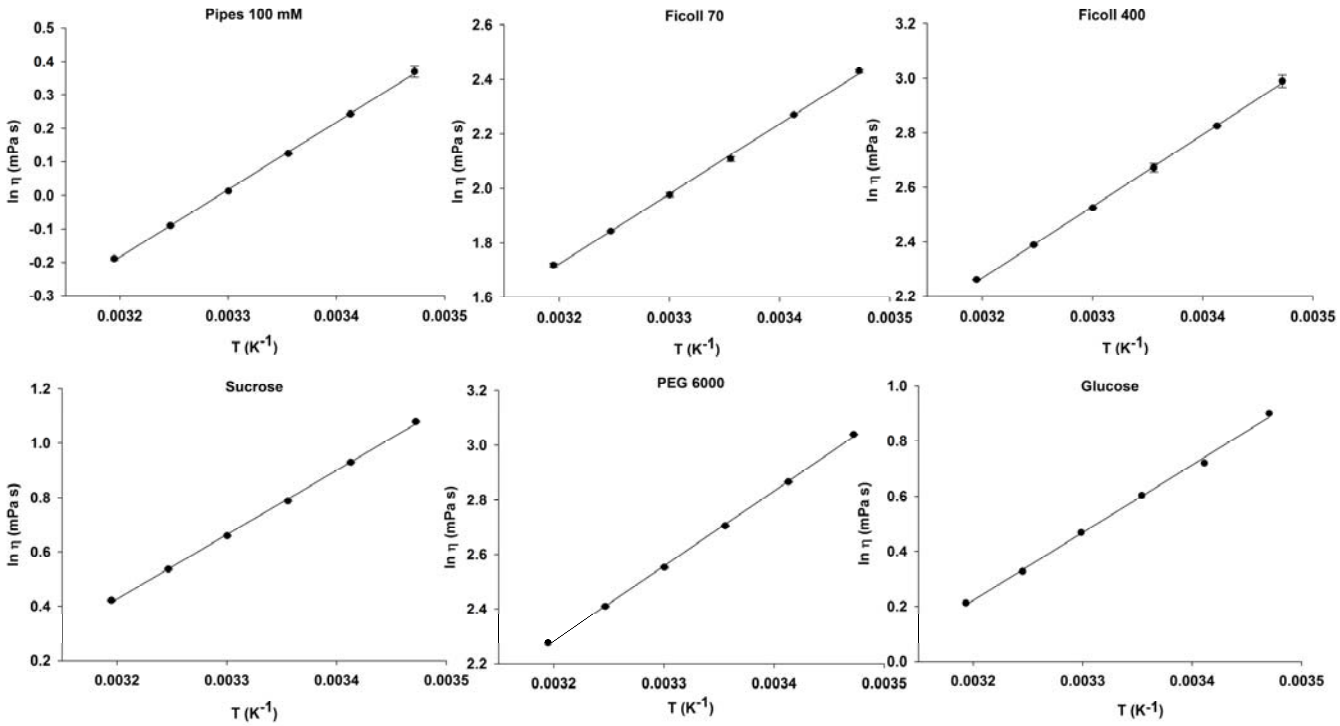

Supplementary Figure SF3. Arrhenius plot for the crowding agents (temperature dependence of *ln η*).

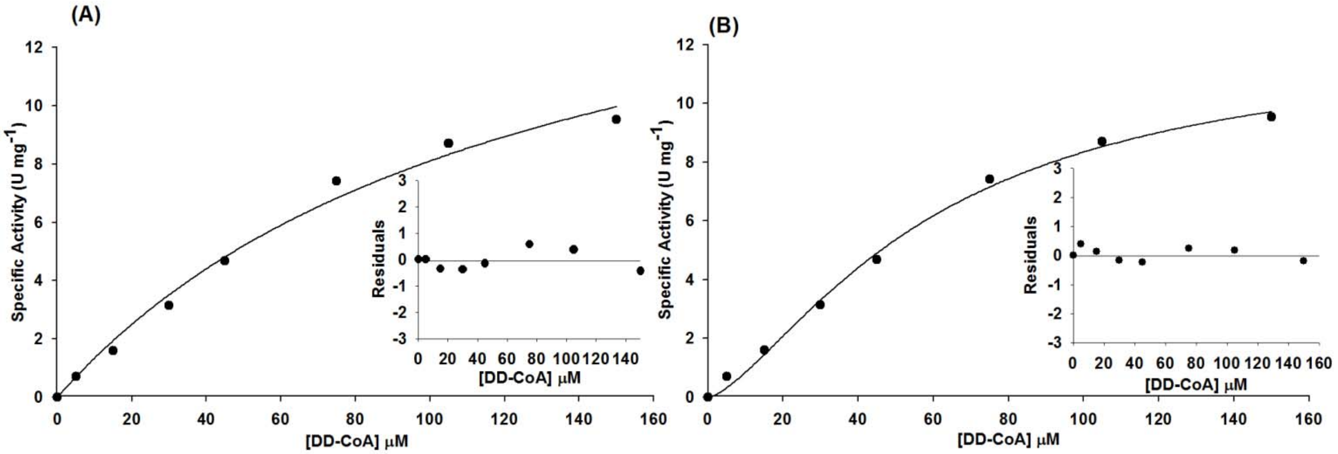

Supplementary Figure SF4. Plot of velocity as a function of increasing DD-CoA concentration in the presence of ficoll 70 at 200 mg mL^{-1}. **(A)** Specific activity (U mg^{-1}) *versus* [DD-CoA] (μM) at fixed concentration of NADH (200 μM), when data were fitted to Michaelis-Menten equation. The inset represents the calculated residual values plotted against DD-CoA concentration using Eq. (1) descibed in the main text. **(B)** Representative plot for specific activity (U mg^{-1}) *versus* [DD-CoA] (μM) at fixed concentration of NADH (200 μM), when data were fitted to the Hill equation. The inset represents the calculated residual values plotted against DD-CoA concentration using Eq. (3) described in the main text.

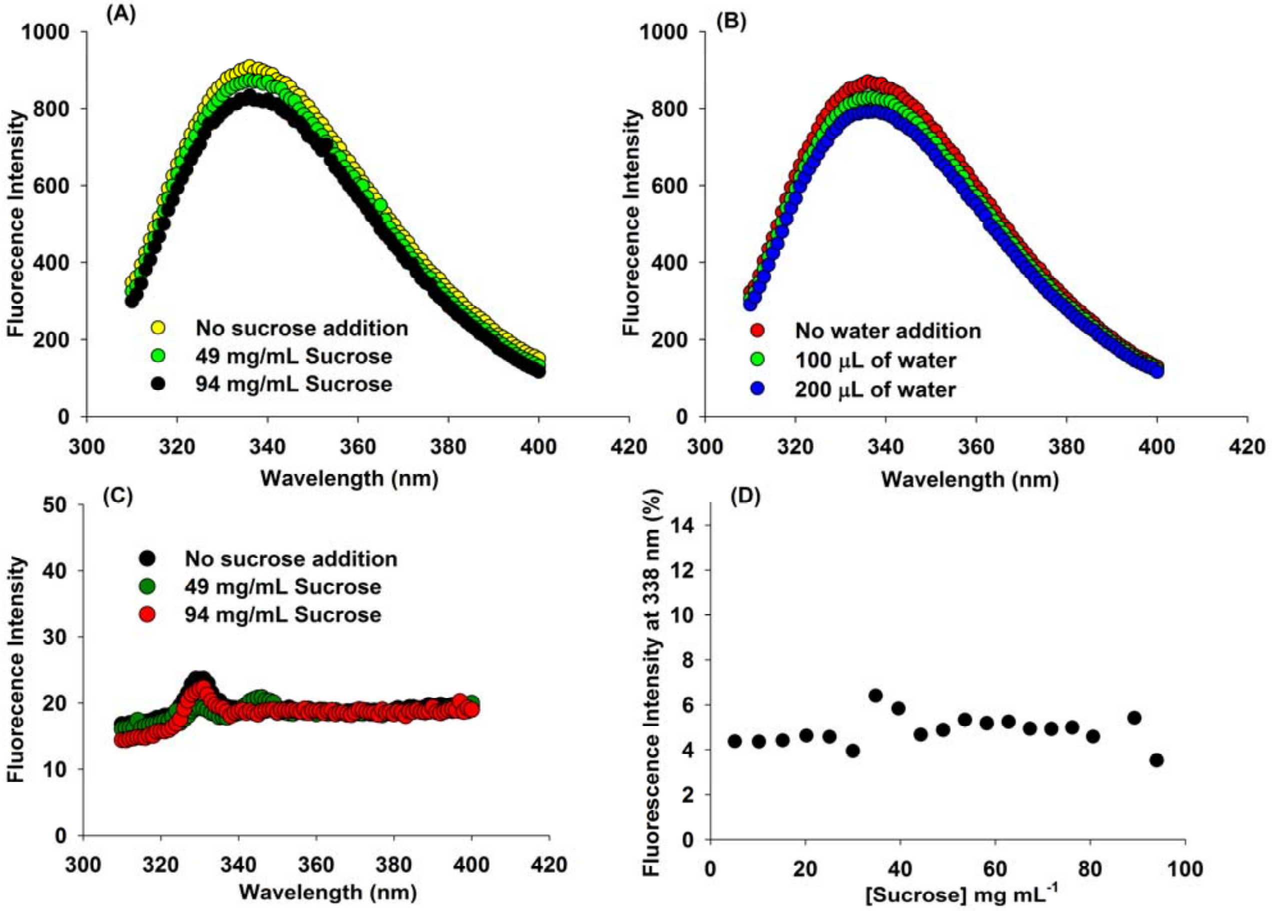

Supplementary Figure SF5. Plots of intrinsic protein fluorescence experiments. (A) Representative fluorescence emission spectra of free InhA (2 μM) and enzyme in solutions containing sucrose. (B) Representative fluorescence emission spectra of InhA (2 μM) upon addition of water to evaluate effects of protein dilution on the enzyme intrinsic protein fluorescence. (C) Representative fluorescence emission spectra of Pipes 100 mM pH 7.0 in solutions containing sucrose. (D) Plot of fluorescence intensity at 338 nm of solutions containing InhA and increasing sucrose concentrations.

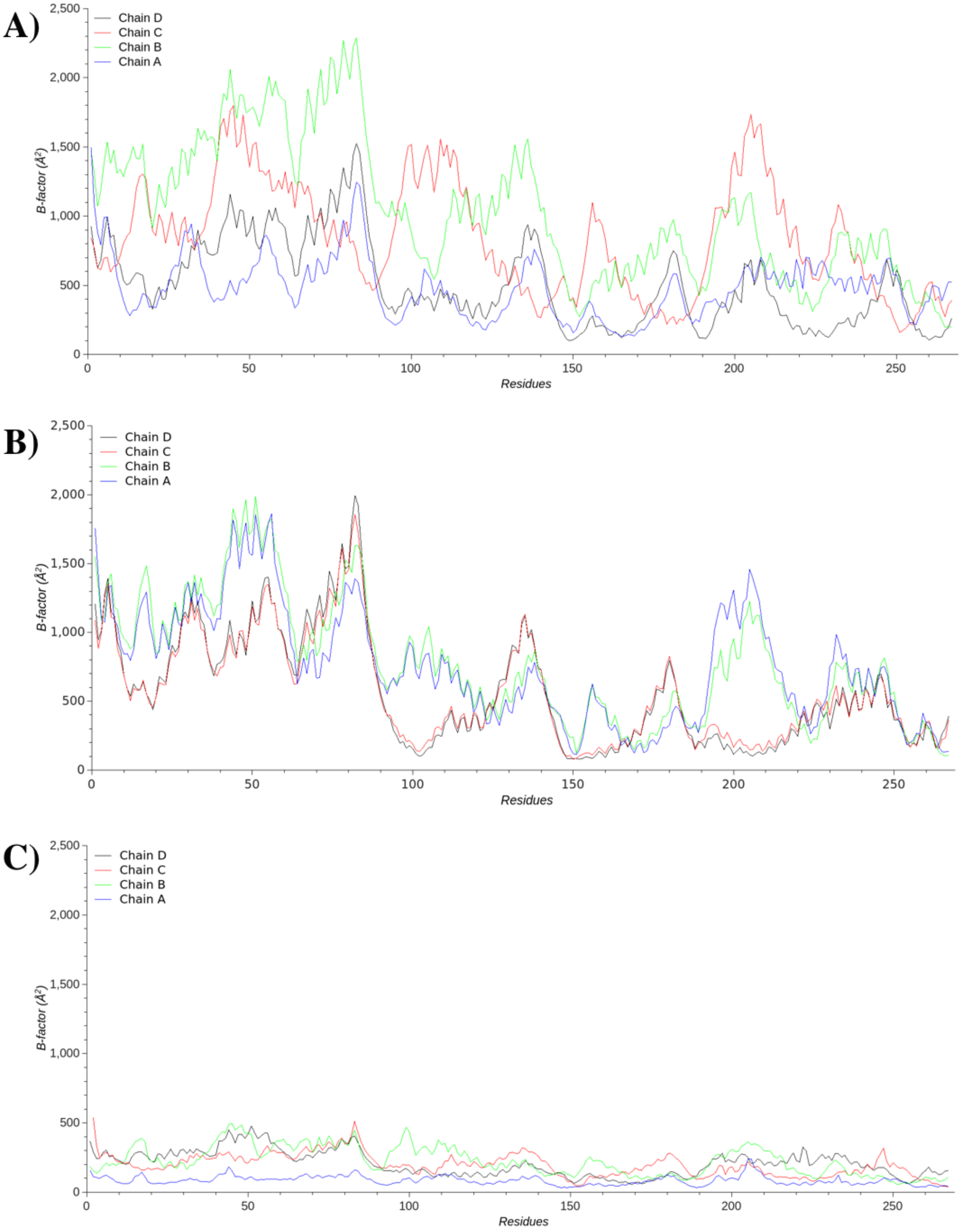

Supplementary Figure SF6. B-factor for all MD simulations of InhA during the production phase. (A) InhA in water, (B) InhA in water and 25 mg mL^{-1} of sucrose, and (C) InhA in water and 200 mg mL^{-1} of sucrose. The same scale was chosen to facilitate visualization of the differences between simulations. Image prepared with QtiPlot.[3]

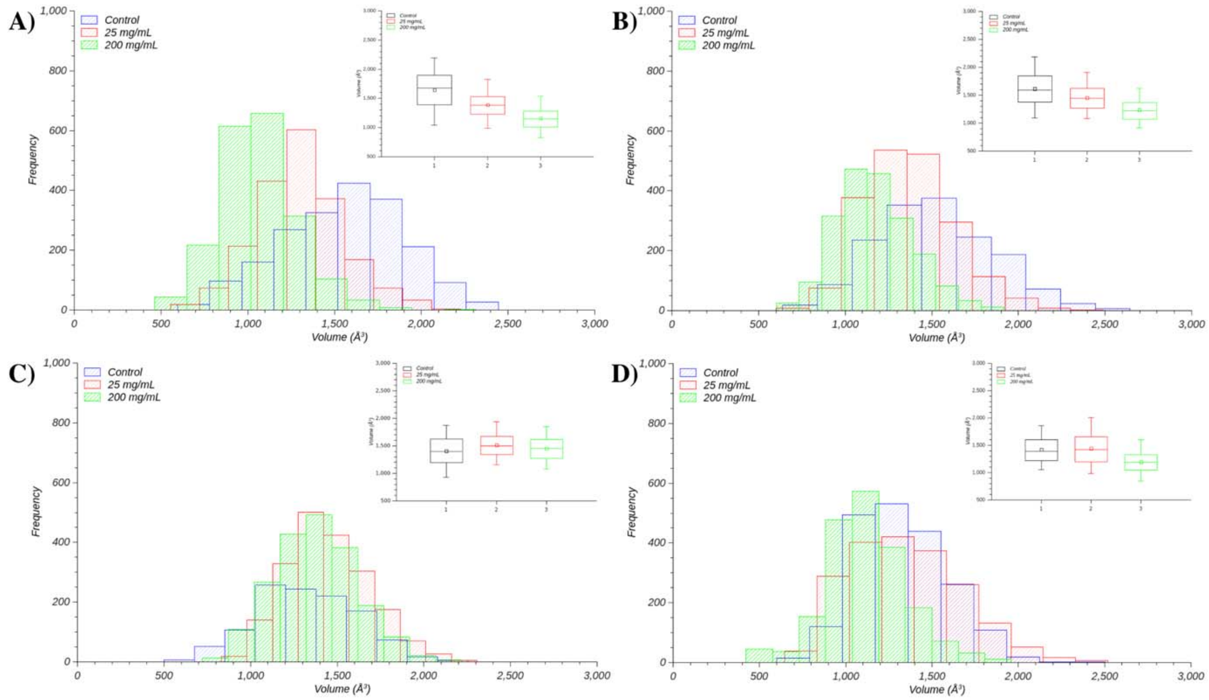

Supplementary Figure SF7. Distribution of the volume of InhA active site cavity. (A) Subunit A, (B) Subunit B, (C) Subunit C, and (D) Subunit D. The control, 25 mg mL^{-1}, and 200 mg mL^{-1} simulations are colored in blue, red and, green, respectively. Top insets show box plots with the mean and variance of the values. Image prepared with QtiPlot.[3]

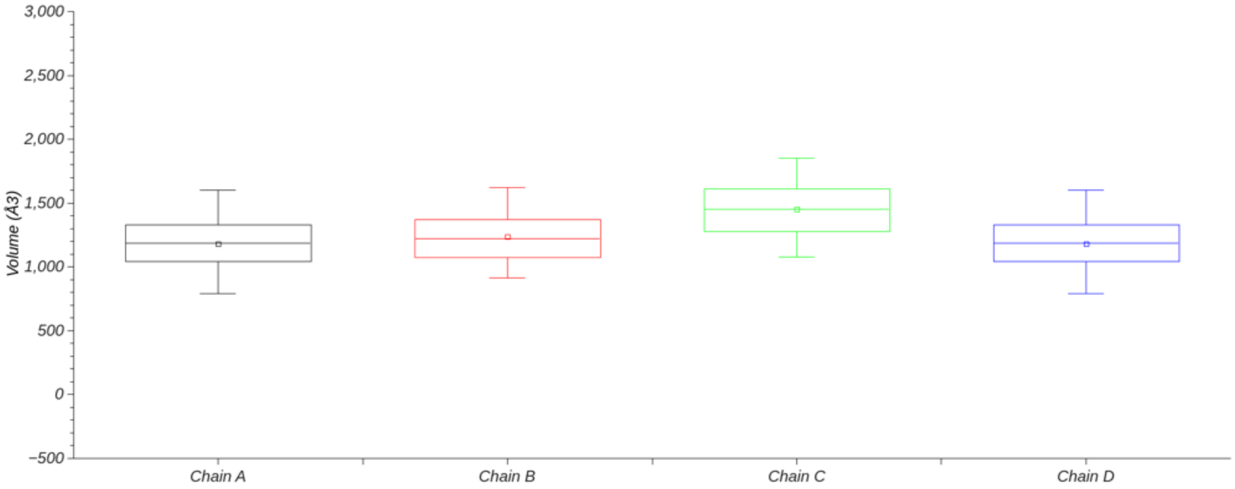

Supplementary Figure SF8. Box plot for the variance of the volume of the volume of InhA active site cavity during the simulation at 200 mg mL^{-1} sucrose concentration. (A) Subunit A in black, (B) Subunit B in red, (C) Subunit C in green, and (D) Subunit D in blue. Image prepared with QtiPlot.[3]

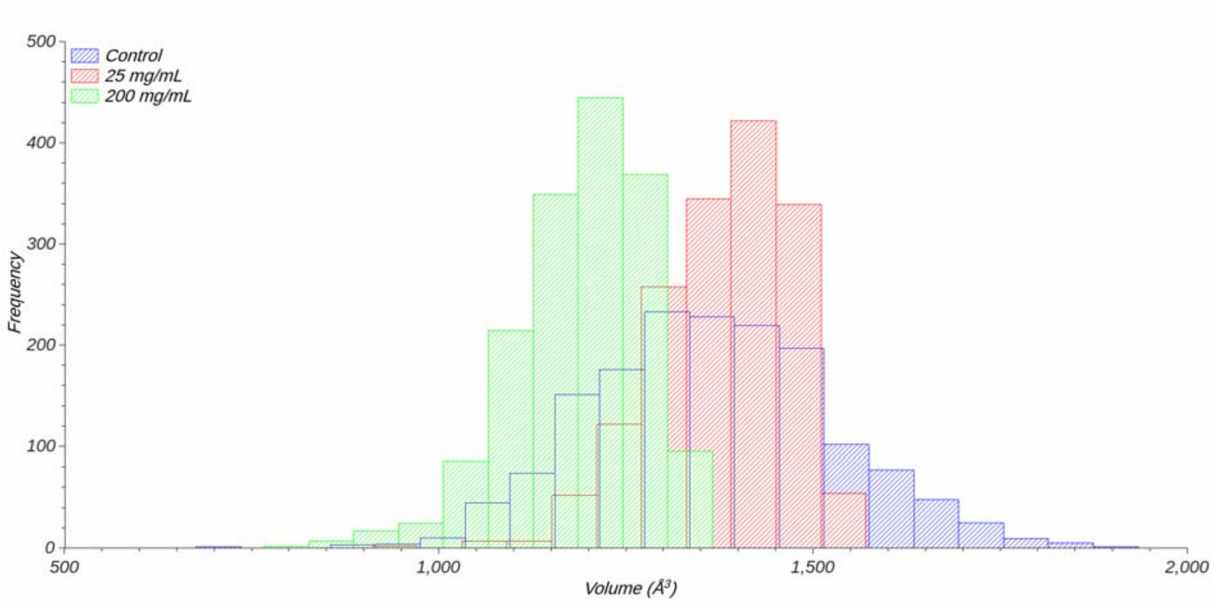

Supplementary Figure SF9. Distribution of active site cavity of InhA in the absence (blue) and in the presence of 25 mg ml^{-1} (red) and 200 mg ml^{-1} (green) of sucrose.

Table ST4. Games-Howell (GH) test using a confidence level of 95%.

Treatment	Subunit	p-value
Control vs. Sucrose_200mg	A	< 0.01
Control vs. Sucrose_200mg	B	< 0.01
Control vs. Sucrose_200mg	C	0.14
Control vs. Sucrose_200mg	D	< 0.01
Control vs. Glucose_200mg	A	1.00
Control vs. Glucose_200mg	B	< 0.01
Control vs. Glucose_200mg	C	1.00
Control vs. Glucose_200mg	D	< 0.01
Sucrose vs. Glucose_200mg	A	< 0.01
Sucrose vs. Glucose_200mg	B	< 0.01
Sucrose vs. Glucose_200mg	C	1.00
Sucrose vs. Glucose_200mg	D	< 0.01

References

(1) Wandrey, C.; Bartkwiak, A.; Hunkeler, D. Partial Molar and Specific Volumes of Polyelectrolytes: Comparison of Experimental and Predicted Values in Salt-free Solutions. Langmuir 1999, 15 (12), 4062-4068.

(2) Halpern, A. M. Experimental Physical Chemistry, Prentice Hall, Inc: New Jersey, 1997, ch. 10, pp. 493-504.

(3) Vasilef, I. QtiPlot: Data Analysis and Scientific Visualization, 2009.
